# Cost benefit analysis of alternative testing and quarantine policies for travelers for infection control: A case study of Singapore during the COVID-19 pandemic

**DOI:** 10.3389/fpubh.2023.1101986

**Published:** 2023-02-23

**Authors:** Jing Lou, Nigel Wei-Han Lim, Celestine Grace XueTing Cai, Borame Sue Lee Dickens, Vinh Anh Huynh, Hwee-Lin Wee

**Affiliations:** ^1^Saw Swee Hock School of Public Health, National University of Singapore, Singapore, Singapore; ^2^Department of Pharmacy, Faculty of Science, National University of Singapore, Singapore, Singapore

**Keywords:** COVID-19, testing, quarantine, travel, cost benefit analysis, border control, infection control, policy

## Abstract

**Background:**

Border control mitigates local infections but bears a heavy economic cost, especially for tourism-reliant countries. While studies have supported the efficacy of border control in suppressing cross-border transmission, the trade-off between costs from imported and secondary cases and from lost economic activities has not been studied. This case study of Singapore during the COVID-19 pandemic aims to understand the impacts of varying quarantine length and testing strategies on the economy and health system. Additionally, we explored the impact of permitting unvaccinated travelers to address emerging equity concerns. We assumed that community transmission is stable and vaccination rates are high enough that inbound travelers are not dissuaded from traveling.

**Methods:**

The number of travelers was predicted considering that longer quarantine reduces willingness to travel. A micro-simulation model predicted the number of COVID-19 cases among travelers, the resultant secondary cases, and the probability of being symptomatic in each group. The incremental net monetary benefit (INB) of Singapore was quantified under each border-opening policy compared to pre-opening status, based on tourism receipts, cost/profit from testing and quarantine, and cost and health loss due to COVID-19 cases.

**Results:**

Compared to polymerase chain reaction (PCR), rapid antigen test (ART) detects fewer imported cases but results in fewer secondary cases. Longer quarantine results in fewer cases but lower INB due to reduced tourism receipts. Assuming the proportion of unvaccinated travelers is small (8% locally and 24% globally), allowing unvaccinated travelers will accrue higher INB without exceeding the intensive care unit (ICU) capacity. The highest monthly INB from all travelers is $2,236.24 m, with 46.69 ICU cases per month, achieved with ARTs at pre-departure and on arrival without quarantine. The optimal policy in terms of highest INB is robust under changes to various model assumptions. Among all cost-benefit components, the top driver for INB is tourism receipts.

**Conclusions:**

With high vaccination rates locally and globally alongside stable community transmission, opening borders to travelers regardless of vaccination status will increase economic growth in the destination country. The caseloads remain manageable without exceeding ICU capacity, and costs of cases are offset by the economic value generated from travelers.

## 1. Introduction

Tourism is a major component of the world economy, contributing 9.8% of global GDP and ranked third largest among the world's exports (1). However, such human movement exacerbates the spread of infectious diseases (2). Border control is critical for reducing the risk of imported cases between highly-connected countries during a pandemic. Epidemiological studies have been investigating how quarantine length and different testing strategies for incoming travelers on arrival affect the effectiveness of imported case detection (3–5). However, the net economic benefits of different testing and quarantine policies are still unclear. Cost-effective border control policies must balance the economic benefits from reduction of case importation risk against the economic burden of testing and quarantine of incoming travelers and the associated reduction in tourism receipts.

At the start of the COVID-19 pandemic, imported cases formed a large proportion of the total incidence worldwide (6). Countries closed their borders, imposed travel restrictions both within and between countries, implemented diagnostic testing strategies and mandated 14–21-day quarantines for incoming travelers. These travel-related control measures reduced the risk of imported cases, but they also cost substantial economic consequences to the global tourism industry and wider society, at an estimated of US$400 billion every month (7).

With the majority of the population eventually vaccinated, countries which initially employed suppression strategies against COVID-19 started relaxing their border control policies and reopening for travel and business, with shorter quarantines and less frequent testing (8). However, border-opening policies are mostly in favor of vaccinated travelers with unvaccinated travelers still required to undergo long quarantines and repeated testing to enter some countries. Having differentiated border policies based on vaccination status creates a social divide and poses ethical concerns (9). These issues arise from the fact that not all countries have access to COVID-19 vaccines, and some individuals are not medically eligible to receive the vaccine (9). While applying similar border control policies for both vaccinated travelers and unvaccinated travelers may improve equity, the latter group is likely to pose economic and epidemiological challenges to society and healthcare system due to their higher susceptibility to infection and severe symptoms.

This cost-benefit analysis uses Singapore in the context of the COVID-19 pandemic as a case study to determine the effects of varying testing and quarantine policies for travelers on monetary benefit and COVID-19 case counts. A model was constructed to allow for different policy combinations (varied quarantine length, test frequencies and type of tests) and to quantify their impacts from a societal perspective. We included four groups of travelers in the model: vaccinated inbound travelers, unvaccinated inbound travelers, vaccinated returning travelers and unvaccinated returning travelers. Inbound travelers and returning travelers influence a country's economy through different mechanisms and were modeled separately. The economic and epidemiological impacts of vaccinated and unvaccinated travelers would determine if it is feasible to allow unvaccinated travelers to enjoy more relaxed border-opening policies, similar to those of vaccinated travelers.

The aims of this cost benefit analysis study are:

To understand the impacts of varying quarantine length and testing strategies at the Singapore border on incoming and returning travelers.
To quantify the epidemiological and economic impacts of allowing unvaccinated individuals to enter Singapore under the same relaxed border-opening policy as vaccinated individuals.

## 2. Methods

### 2.1. Population and subgroups

The population of interest consists of inbound travelers and returning travelers to Singapore, as well as the Singapore resident population which is susceptible to infection. Returning travelers include Singapore citizens and residents (permanent, work or visit pass holders) who traveled out of Singapore and then returned.

### 2.2. Setting

The setting for this analysis is a healthcare system prepared to accommodate imported COVID-19 cases and associated secondary cases, serving a local general population with high and stable COVID-19 vaccination rates. Given that the maximum number of ICU beds in Singapore is ~500 (10), we set aside a maximum of 100 ICU beds for inbound and returning travelers and the resulting secondary cases. The border-opening policy (a quarantine length and a testing strategy) was assumed to be the same for both inbound travelers and returning travelers, regardless of vaccination status.

### 2.3. Study perspective

Societal perspective of Singapore.

### 2.4. Comparators

Eleven testing strategies were examined (Table 1), each with a quarantine length ranging from 0 to 14 days. Testing strategies varied in types of test, between antigen rapid test (ART) and polymerase chain reaction (PCR), and also in testing frequency (whether to test, including or excluding compulsory exit test at the end of quarantine, and frequency of tests during quarantine). Pre-departure test, test upon arrival at Singapore border (entry test) and test at the end of quarantine (exit test) could be either ART or PCR, while tests during quarantine are all ART across testing strategies. In the base case, the processing time for PCR is assumed to be 1 day. Hence, the length of quarantine is at least 1 day when PCR is used for the entry test. Each policy analysis consists of a combination of one quarantine length and one testing strategy. For example, a 7-day quarantine with testing strategy S6 consists of a pre-departure test, entry test and exit test using PCR and a daily test during quarantine using ART.

**Table 1:** Testing strategies.

**Strategy Number**	Type of test^*^	**Pre-departure test**	**Entry test**	**Exit test**	**Tests during quarantine**		
					**Every 7 days** ^**^	**Every 3 days** ^**^	**Daily** ^**^
**S1**	ART	✓	✓	✓			✓
**S2**	ART	✓	✓	✓		✓	
**S3**	ART	✓	✓	✓	✓		
**S4**	ART	✓	✓	✓			
**S5**	ART	✓	✓				
**S6**	PCR	✓	✓	✓			✓
**S7**	PCR	✓	✓	✓		✓	
**S8**	PCR	✓	✓	✓	✓		
**S9**	PCR	✓	✓	✓			
**S10**	PCR	✓	✓				
**S11**	NA	Quarantine only					

^*^PCR tests assumed to have a processing time of 1 day in base case analysis. Quarantine length is at least 1 day when implementing S6-S10.

^**^ART by default.

ART, antigen rapid test; PCR, polymerase chain reaction; S1, …, S11, eleven testing strategies varying in type and frequency of test.

### 2.5. Time horizon

The model used a 1 month time horizon. Specifically, for each border-opening policy, we predicted the numbers of vaccinated and unvaccinated, inbound and returning travelers within a month. For each subgroup of travelers, all imported cases among them and all secondary cases caused by them were counted, recoveries and secondary infections beyond 1 month included.

### 2.6. Discount rate

Discounting is not applicable given the 1 month time horizon, as all expenses are expected to be incurred within a few days to a few months (in case of long COVID). The exception is health loss from pre-mature death, which is calculated as a lifetime cost with an annual discount rate of 3% (11).

### 2.7. Measurement of effectiveness

The effectiveness of a border-opening policy is measured by the estimated numbers of imported cases and associated secondary cases, as well as the number of ICU cases and death toll. Case counts among inbound travelers, returning travelers and secondary cases from each group of travelers are reported separately. To evaluate whether a border-opening policy provides good disease control, we compared the monthly total number of patient-days in ICU with the cut-off value of 3,000, based on 100 ICU beds (the estimated maximum number of ICU beds that could be prepared for international transmissions) for 30 days in a month. The monthly total number of patient-days in ICU equals the monthly number of vaccinated ICU cases, multiplied by 6 days in ICU per case, plus the monthly number of unvaccinated ICU cases, multiplied by 10 days in ICU per case. The disease period data was provided by Singapore's Ministry of Health (MOH).

### 2.8. Choice and evaluation of outcomes

Health outcomes included COVID-19-related morbidity and mortality. Quality adjusted life year (QALY) losses were first derived based on estimates of health-related quality of life (HRQoL) scores of COVID-19 patients and probability and duration of long-COVID (12–14). Then the QALY losses were translated into Singapore dollars by multiplication with the cost-effectiveness threshold (CET), assumed to be $7500 in the base case analysis, an implicit threshold based on a review of appraisals by Agency of Care Effectiveness Singapore (15).

As a cost-benefit analysis, the outcome of interest is the incremental net benefit (INB) of Singapore under each border-opening policy compared to that under the pre-opening policy, a 14-day quarantine with PCRs pre-departure, upon arrival and at the end of quarantine, with weekly ART during quarantine (S8 in Table 1).

### 2.9. Currency, price date and conversion

The currency used is Singapore dollars (S$). The exchange rate in June 2022 ranged from US$0.7174 to US$0.7297 per S$1 (16). The study was carried out from 2021 to 2022. The price data was collected in August and September 2021, and in June 2022.

### 2.10. Model design

For a given testing and quarantine policy, the numbers of inbound and returning travelers were predicted whilst taking into account that longer quarantine reduces willingness to travel (17). A transmission sub-model predicted the number of COVID-19 cases among these travelers, the secondary cases that resulted, and the probability of having symptomatic disease in each group. Lastly, based on the numbers of travelers and COVID-19 cases, the net monetary benefit (NMB) of Singapore was quantified under each border-opening policy, taking into account tourism receipts, cost/profit from testing and quarantine, and cost and health loss due to COVID-19 cases. The values and data sources of model parameters are available in Supplementary material 1.

The monthly numbers of travelers without quarantine were assumed to be the same as pre-COVID: 1.53 million inbound travelers and 0.86 million returning travelers, based on monthly numbers of travelers by air and sea recorded by Singapore Tourism Board (STB) (18) from January 2017 to January 2020. The monthly number of travelers under a 14-day quarantine was assumed to be at the same level as from January to August 2021, when such a quarantine was required by most countries at their border: 0.02 million inbound travelers and 0.05 million returning travelers. Assuming a constant percentage reduction in the number of travelers with each extra day of quarantine, the number of inbound travelers decreases by 14.49%, and the number of returning travelers decreases by 9.66%. The base case analysis assumed that the length of quarantine when entering countries in the rest of the world (ROW) is the same as that for Singapore, as bilateral policies are typically reciprocal. Vaccine coverage (minimally received 2 doses of WHO approved COVID-19 vaccine) was assumed to be 92% in Singapore and 76% in the ROW (as of June 2022) (19, 20). The ratio of the number of vaccinated travelers to the number of unvaccinated travelers was 92:8 among returning travelers and 76:24 among inbound travelers for modeling scenarios wherein vaccinated and unvaccinated travelers were subjected to the same testing and quarantine policy options.

The transmission sub-model uses Monte Carlo simulation to estimate COVID-19 transmission outcomes for travelers to Singapore from overseas under each policy option of testing and quarantine. Vaccinated and unvaccinated, inbound and returning travelers were modeled separately. For each traveler group, travelers who yield positive test results or develop symptoms before the end of quarantine, or recover within quarantine, are removed from the infectious group (tabulated as detected pre-departure or at the Singapore border). The remaining infectious travelers exit quarantine and contribute to secondary transmission in Singapore until they recover or leave Singapore. Imported cases (either detected at the Singapore border or in the community by tests or symptoms) and secondary cases caused by travelers were quantified. The risk stratification of imported cases and secondary cases were estimated based on the age structure of each group (18, 21), vaccination coverage (20) and vaccine efficacy against the Omicron variant (22, 23). Compared to vaccinated travelers, unvaccinated travelers have higher COVID-19 prevalence, higher rates of ICU utilization and higher chances of being detected, especially through symptomatic infections.

The transmission sub-model extends previous work by Dickens et al. (3, 4). It incorporates the effects of vaccination, as well as differences in distributions of infection-related statistics (particularly the day of infection) between inbound and returning travelers. The R0, prevalence rate, risk profile and the viral load profile used to derive test sensitivities have been updated for the Omicron variant. More details about the transmission sub-model are provided in Supplementary material 2. Model parameters and data sources are provided in Supplementary material 1.

With the numbers of travelers and COVID-19 cases estimated under each border-opening policy, the NMB and INB (compared to pre-opening status of January–August 2021) of Singapore were quantified for each traveler group. The model assumed that inbound travelers who are diagnosed with COVID-19 on arrival or during quarantine will bear all treatment costs. Inbound travelers' expenditure on quarantine was assumed to constitute a net economic gain for hotels in Singapore, as empty hotels are a sunk cost and hotel staff would be unemployed if there were no travelers. Quarantine for returning travelers only incurred productivity loss to the travelers as payment for hotel may be considered as a transfer from one segment of Singapore society (individuals) to another segment (hotels). Inbound travelers' expenditure on tests minus the cost of the tests was considered a net economic gain (or profit) to Singapore. Returning travelers' expenditure on tests was considered a cost to Singapore. Costs were derived from publicly available data by Singapore's local authorities and healthcare providers. Healthcare costs incurred by returning travelers overseas, including pre-departure tests for every traveler and treatment costs for the detected cases, were estimated by rescaling the costs in Singapore with a factor of 35.78%. We obtained this factor by taking a weighted average of the ratios of per capita health expenditure (PCHE) for the main destination countries of Singaporean travelers' relative to PCHE for Singapore (24). Weights of each main destination were based on the proportion of Singaporean travelers to the country (25). More details about economic quantification are provided in Supplementary material 3. Model parameters and data sources are provided in Supplementary material 1.

The components of the NMB differ between inbound travelers and returning travelers (Supplementary Table A.7, Supplementary material 3). The cost components from inbound travelers include the direct costs, productivity losses and health losses (in monetary terms) from secondary cases caused by inbound travelers, as well as the cost of testing close contacts of secondary cases. The benefit components from inbound travelers include tourism receipts and profits from quarantining and testing them. The cost components from returning travelers include costs from quarantining and testing them, the direct costs, productivity losses and health losses (in monetary terms) to returning travelers themselves and from secondary cases caused by them, as well as the cost of testing close contacts of the secondary cases. Returning travelers were assumed costs and no benefits for Singapore.

### 2.11. Statistical analysis

Model parameters were estimated using statistical methods such as taking the mean average, maximum and minimum of multiple data points; calibrating parameters based on observational data. More details are provided in Supplementary material 1, 2.

### 2.12. Sensitivity analysis

A one-way deterministic sensitivity analysis was conducted. First, the percentage reductions in the number of travelers for each extra day of quarantine (14.49% for inbound travelers and 9.66% for returning travelers for base case analysis) were increased and decreased 0.5-fold. Second, the length of quarantine to enter countries in the ROW was fixed at 0 days or 14 days while the length of quarantine to enter Singapore varied across 0–14 days.

Third, we fixed the COVID-19 prevalence rates in Singapore and ROW at their maximal and minimal values over the period from January to the middle of June 2022. The base case analysis used the average prevalence rates over this period. Higher prevalence rates may require more stringent testing and quarantine policies to achieve a higher NMB and vice versa. Fourth, we increased the R0 of Omicron from 8.2 (26) to a high estimate of 10 (27) and an assumed value of 15 to model the possibility of a new variant. Fifth, we reduced the vaccination coverage from 76% in ROW and 92% in SG, which was the coverage for having received the full regimen plus a booster shot or full regimen only as of June 2022, to 30% in ROW and 78% in SG, which was the coverage of having received the full regimen plus a booster shot as of June 2022 (19, 20). When vaccination programs become non-mandatory and out-of-pocket payment is required for vaccination, the vaccination coverage may be lower than the current status. Sixth, the assumed efficacy of vaccines among inbound travelers was lowered from the efficacy of mRNA vaccine (base case analysis) to the efficacy of the inactivated vaccine (28). Seventh, we increased the PCR processing time to 2 days. An increase in processing time is likely if the daily PCR testing capacity is overwhelmed by a large number of travelers.

Eighth, we incorporated the tourism multiplier effect, as receipts in the tourism sector will likely have spill-over effect on upstream sectors (29). This spill-over effect is quantified by a multiplier derived using Leontief's matrix based on Singapore's Input-Output Table (21) and historical tourism receipt components during 2016–2020 (18). Ninth, we reduced the productivity loss due to quarantine to 0%, which may be plausible since returning travelers may still work remotely while in quarantine or use their annual leave. Tenth, the healthcare expenditure levels in destination countries relative to Singapore were varied from a lower bound of 0.04 (PCHE of Indonesia relative to Singapore) to an upper bound of 1.90 (PCHE of Australia relative to Singapore) among the 7 major destination countries for Singaporean travelers (24, 25). Eleventh, we doubled the estimated medical costs of COVID-19 cases. Twelfth, the QALY losses due to morbidity for each type of symptomatic case were matched to high estimates from literature (30) on other respiratory diseases. Thirteenth, the CET of Singapore was varied from $39199 (0.4 times of GDP per capita, to proxy a supply-side CET) to $293,394 (3 times of GDP per capita) (21, 31, 32), allowing for lower and higher economic impacts of health loss due to COVID-19 morbidity and mortality.

## 3. Results

### 3.1. Impacts of testing

The epidemiological performance of testing strategies does not differ across traveler groups (Tables 2, 3). Increasing the frequency of testing picks up more imported cases and lowers the number of secondary cases. The number of ICU cases and deaths among travelers are not affected by frequency of tests during quarantine or including / excluding quarantine exit test, because cases will be detected as soon as they develop systems. PCR detects more imported cases than ART does, due to higher sensitivity of PCR (33, 34). However, ART results in fewer secondary cases, as well as fewer ICU cases and deaths among secondary cases. This is due to the 1-day processing delay of PCR (4, 35).

**Table 2:** Monthly COVID-19 case counts and INB from vaccinated travelers under different strategies.

	**Quarantine Length**																							
	**0 days**								**7 days**								**14 days**							
	**Travelers**			**Community**			**INB (in millions)**	**Total INB (in millions)**	**Travelers**			**Community**			**INB (in millions)**	**Total INB (in millions)**	**Travelers**			**Community**			**INB (in millions)**	**Total INB (in millions)**
**Testing strategy**	**Imported cases**	**ICU cases**	**Deaths**	**Secondary cases**	**ICU cases**	**Deaths**			**Imported cases**	**ICU cases**	**Deaths**	**Secondary cases**	**ICU cases**	**Deaths**			**Imported cases**	**ICU cases**	**Deaths**	**Secondary cases**	**ICU cases**	**Deaths**		
							**Inbound Travelers**																								
							**S1**	–							–	–							–	–	–	–	–	159.88	0.64	0.16	17.41	0.07	0.03	$322.48	$286.83	18.37	0.07	0.02	0.40	0.00	0.00	–$0.33	$1.48
							**S2**	–							–	–							–	–	–	–	–	159.10	0.64	0.16	17.54	0.07	0.03	$318.14	$285.10	18.25	0.07	0.02	0.42	0.00	0.00	–$1.42	$1.04
**S3**	–	–	–	–	–	–	–	–	153.86	0.64	0.16	24.47	0.09	0.04	$315.95	$284.20	17.26	0.07	0.02	0.58	0.00	0.00	–$1.78	$0.90
**S4**	–	–	–	–	–	–	–	–	147.92	0.64	0.16	28.84	0.11	0.05	$314.85	$283.75	14.44	0.07	0.02	1.26	0.00	0.00	–$2.03	$0.80
**S5**	1,122.67	5.70	1.45	1245.44	4.80	2.07	$1,687.81	$1,707.23	125.46	0.64	0.16	58.71	0.23	0.10	$313.48	$282.97	14.02	0.07	0.02	1.80	0.01	0.00	–$2.18	$0.72
**S6**	–	–	–	–	–	–	–	–	172.30	0.64	0.16	18.44	0.07	0.03	$338.44	$278.78	19.79	0.07	0.02	0.42	0.00	0.00	$1.46	$0.58
**S7**	–	–	–	–	–	–	–	–	172.05	0.64	0.16	18.64	0.07	0.03	$334.10	$277.04	19.72	0.07	0.02	0.48	0.00	0.00	$0.36	$0.15
**S8**	–	–	–	–	–	–	–	–	168.60	0.64	0.16	24.90	0.10	0.04	$331.91	$276.12	18.74	0.07	0.02	0.59	0.00	0.00	$0.00	$0.00
**S9**	–	–	–	–	–	–	–	–	163.54	0.64	0.16	29.45	0.11	0.05	$330.81	$275.67	15.58	0.07	0.02	1.08	0.00	0.00	–$0.24	–$0.10
**S10**	–	–	–	–	–	–	–	–	133.11	0.64	0.16	65.62	0.25	0.11	$322.58	$275.56	14.87	0.07	0.02	1.80	0.01	0.00	–$1.16	–$0.10
**S11**	664.78	6.86	1.74	2,144.36	8.27	3.56	$1,674.75	$1,700.55	74.29	0.77	0.19	83.27	0.32	0.14	$312.31	$282.70	8.30	0.09	0.02	1.80	0.01	0.00	–$2.29	$0.71
**Returning travelers**																								
**S1**	–	–	–	–	–	–	–	–	165.03	0.24	0.07	68.32	0.26	0.11	–$35.65	$286.83	39.80	0.06	0.02	3.03	0.01	0.01	$1.81	$1.48
**S2**	–	–	–	–	–	–	–	–	165.02	0.24	0.07	68.45	0.26	0.11	–$33.04	$285.10	39.80	0.06	0.02	3.21	0.01	0.01	$2.46	$1.04
**S3**	–	–	–	–	–	–	–	–	165.01	0.24	0.07	74.31	0.29	0.12	–$31.75	$284.20	39.80	0.06	0.02	3.25	0.01	0.01	$2.68	$0.90
**S4**	–	–	–	–	–	–	–	–	164.99	0.24	0.07	74.34	0.29	0.12	–$31.10	$283.75	39.78	0.06	0.02	3.25	0.01	0.01	$2.83	$0.80
**S5**	683.87	0.99	0.28	1,971.85	7.61	3.27	$19.43	$1,707.23	164.93	0.24	0.07	95.84	0.37	0.16	–$30.51	$282.97	39.78	0.06	0.02	4.03	0.02	0.01	$2.90	$0.72
**S6**	–	–	–	–	–	–	–	–	167.39	0.26	0.07	72.68	0.28	0.12	–$59.66	$278.78	40.37	0.06	0.02	3.19	0.01	0.01	–$0.87	$0.58
**S7**	–	–	–	–	–	–	–	–	167.39	0.26	0.07	73.13	0.28	0.12	–$57.05	$277.04	40.37	0.06	0.02	3.68	0.01	0.01	–$0.22	$0.15
**S8**	–	–	–	–	–	–	–	–	167.38	0.26	0.07	88.77	0.34	0.15	–$55.79	$276.12	40.37	0.06	0.02	3.79	0.01	0.01	$0.00	$0.00
**S9**	–	–	–	–	–	–	–	–	167.36	0.26	0.07	88.80	0.34	0.15	–$55.14	$275.67	40.34	0.06	0.02	3.79	0.01	0.01	$0.15	–$0.10
**S10**	–	–	–	–	–	–	–	–	167.27	0.26	0.07	95.87	0.37	0.16	–$47.02	$275.56	40.34	0.06	0.02	4.03	0.02	0.01	$1.05	–$0.10
**S11**	721.74	1.12	0.28	2,575.51	9.94	4.27	$25.80	$1,700.55	174.06	0.27	0.07	95.93	0.37	0.16	–$29.61	$282.70	41.98	0.07	0.02	4.03	0.02	0.01	$3.00	$0.71

The monthly case counts and INB of three quarantine length (0 day, 7 days, 14 days) and all testing strategies (S1 - S11 as in Table 1) are displayed.

Numbers in the upper panel are all from vaccinated inbound travelers, and numbers in the lower panel are all from vaccinated returning travelers, except column “Total INB” combines INB from vaccinated inbound and vaccinated returning travelers.

Column “imported cases” counts the number of vaccinated inbound or vaccinated returning travelers detected by positive test results or symptoms within Singapore border (excluding those detected pre-departure). The numbers of ICU cases and deaths among the imported cases are provided in the next two columns.

Column “secondary cases” counts the number of secondary cases in the community caused by vaccinated inbound or vaccinated returning travelers. The numbers of ICU cases and deaths among the secondary cases are provided in the next two columns.

Column “INB (in millions)” provides the monthly INB (compared to pre-opening period) from vaccinated inbound or vaccinated returning travelers alone.

COVID-19, coronavirus disease 2019; INB, incremental net benefit, compared to pre-opening period; ICU, intensive care unit.

**Table 3:** Monthly COVID-19 case counts and INB from vaccinated and unvaccinated travelers under different strategies.

	**Quarantine Length**																							
	**0 days**								**7 days**								**14 days**							
	**Travelers**			**Community**			**INB (in millions)**	**Total INB (in millions)**	**Travelers**			**Community**			**INB (in millions)**	**Total INB (in millions)**	**Travelers**			**Community**			**INB (in millions)**	**Total INB (in millions)**
**Testing strategy**	**Imported cases**	**ICU cases**	**Deaths**	**Secondary cases**	**ICU cases**	**Deaths**			**Imported cases**	**ICU cases**	**Deaths**	**Secondary cases**	**ICU cases**	**Deaths**			**Imported cases**	**ICU cases**	**Deaths**	**Secondary cases**	**ICU cases**	**Deaths**		
							**Inbound travelers**																								
							**S1**	–							–	–							–	–	–	–	–	275.01	2.58	0.66	30.84	0.12	0.05	$424.24	$384.45	31.52	0.29	0.07	0.70	0.00	0.00	–$0.43	$1.94
							**S2**	–							–	–							–	–	–	–	–	273.80	2.58	0.66	31.06	0.12	0.05	$418.53	$382.16	31.35	0.29	0.07	0.75	0.00	0.00	–$1.87	$1.36
**S3**	–	–	–	–	–	–	–	–	265.51	2.58	0.66	42.29	0.16	0.07	$415.64	$380.96	29.78	0.29	0.07	1.00	0.00	0.00	–$2.35	$1.17
**S4**	–	–	–	–	–	–	–	–	256.08	2.58	0.66	49.24	0.19	0.08	$414.20	$380.37	25.31	0.29	0.07	2.07	0.01	0.00	–$2.67	$1.05
**S5**	1,973.14	23.12	5.87	2,166.22	8.36	3.59	$2,219.05	$2,236.24	220.49	2.58	0.66	97.46	0.38	0.16	$412.36	$379.27	24.64	0.29	0.07	2.94	0.01	0.00	–$2.86	$0.95
**S6**	–	–	–	–	–	–	–	–	295.13	2.65	0.67	32.59	0.13	0.05	$445.25	$373.88	33.82	0.30	0.08	0.74	0.00	0.00	$1.92	$0.77
**S7**	–	–	–	–	–	–	–	–	294.77	2.65	0.67	32.91	0.13	0.05	$439.53	$371.59	33.73	0.30	0.08	0.85	0.00	0.00	$0.48	$0.19
**S8**	–	–	–	–	–	–	–	–	289.28	2.65	0.67	43.58	0.17	0.07	$436.65	$370.36	32.16	0.30	0.08	1.03	0.00	0.00	$0.00	$0.00
**S9**	–	–	–	–	–	–	–	–	281.27	2.65	0.67	50.88	0.20	0.08	$435.20	$369.77	27.17	0.30	0.08	1.81	0.01	0.00	–$0.32	–$0.12
**S10**	–	–	–	–	–	–	–	–	233.21	2.65	0.67	108.34	0.42	0.18	$424.35	$369.54	26.06	0.30	0.08	2.94	0.01	0.00	–$1.52	–$0.13
**S11**	1,262.24	29.22	7.42	3,647.26	14.07	6.05	$2,201.38	$2,226.88	141.05	3.27	0.83	136.21	0.53	0.23	$410.88	$379.03	15.76	0.36	0.09	2.94	0.01	0.00	–$3.00	$0.98
**Returning travelers**																								
**S1**	–	–	–	–	–	–	–	–	230.78	1.19	0.38	92.14	0.36	0.15	–$39.79	$384.45	56.05	0.29	0.09	4.10	0.02	0.01	$2.37	$1.94
**S2**	–	–	–	–	–	–	–	–	230.71	1.19	0.38	92.36	0.36	0.15	–$36.36	$382.16	56.01	0.29	0.09	4.36	0.02	0.01	$3.23	$1.36
**S3**	–	–	–	–	–	–	–	–	228.76	1.19	0.38	103.49	0.40	0.17	–$34.68	$380.96	55.19	0.29	0.09	4.57	0.02	0.01	$3.52	$1.17
**S4**	–	–	–	–	–	–	–	–	227.12	1.19	0.38	105.70	0.41	0.18	–$33.83	$380.37	53.04	0.29	0.09	5.17	0.02	0.01	$3.72	$1.05
**S5**	906.21	4.93	1.56	2,664.76	10.28	4.42	$17.18	$2,236.24	218.55	1.19	0.38	152.59	0.59	0.25	–$33.09	$379.27	52.71	0.29	0.09	6.83	0.03	0.01	$3.81	$0.95
**S6**	–	–	–	–	–	–	–	–	234.14	1.30	0.38	97.91	0.38	0.16	–$71.36	$373.88	56.88	0.31	0.09	4.32	0.02	0.01	–$1.15	$0.77
**S7**	–	–	–	–	–	–	–	–	234.11	1.30	0.38	98.56	0.38	0.16	–$67.94	$371.59	56.84	0.31	0.09	4.98	0.02	0.01	–$0.29	$0.19
**S8**	–	–	–	–	–	–	–	–	232.81	1.30	0.38	121.36	0.47	0.20	–$66.29	$370.36	56.02	0.31	0.09	5.22	0.02	0.01	$0.00	$0.00
**S9**	–	–	–	–	–	–	–	–	231.07	1.30	0.38	123.50	0.48	0.20	–$65.43	$369.77	53.50	0.31	0.09	5.64	0.02	0.01	$0.20	–$0.12
**S10**	–	–	–	–	–	–	–	–	219.54	1.30	0.38	155.71	0.60	0.26	–$54.80	$369.54	52.95	0.31	0.09	6.83	0.03	0.01	$1.39	–$0.13
**S11**	899.12	6.13	1.56	3,597.41	13.88	5.97	$25.50	$2,226.88	216.84	1.48	0.38	161.72	0.62	0.27	–$31.84	$379.03	52.29	0.36	0.09	6.83	0.03	0.01	$3.98	$0.98

The monthly case counts and INB of three quarantine length (0 day, 7 days, 14 days) and all testing strategies (S1–S11 as in Table 1) are displayed.

Numbers in the upper panel are all from inbound travelers, and numbers in the lower panel are all from returning travelers, except column “Total INB” combines INB from inbound and returning travelers.

Column “imported cases” counts the number of inbound or returning travelers detected by positive test results or symptoms within Singapore border (excluding those detected pre-departure). The numbers of ICU cases and deaths among the imported cases are provided in the next two columns.

Column “secondary cases” counts the number of secondary cases in the community caused by inbound or returning travelers. The numbers of ICU cases and deaths among the secondary cases are provided in the next two columns.

Column “INB (in millions)” provides the monthly INB (compared to pre-opening period) from inbound or returning travelers alone.

COVID-19, coronavirus disease 2019; INB, incremental net benefit, compared to pre-opening period; ICU, intensive care unit.

The economic impacts of testing strategies differ between inbound travelers (upper panels of Tables 2, 3) and returning travelers (lower panels of Tables 2, 3). INB from inbound travelers is higher when using PCR and when the testing frequency is higher, due to higher profits from testing inbound travelers. INB from returning travelers is higher when using ART and when the testing frequency is lower, due to lower testing costs of returning travelers.

### 3.2. Impacts of quarantine

Similar to testing, the epidemiological impacts of quarantine length do not differ across traveler groups (Tables 2, 3), while the economic impacts of quarantine length differ between inbound travelers (upper panels of Tables 2, 3) and returning travelers (lower panels of Tables 2, 3). Regardless of traveler group, longer quarantine results in fewer cases, because (i) a longer quarantine reduces willingness to travel, (ii) cases are more likely to recover before the end of a longer quarantine, and (iii) with a fixed testing frequency, a longer quarantine will allow more tests and reduce the number of missed cases. A longer quarantine results in lower INB from inbound travelers due to fewer travelers bringing in tourism receipts. In contrast, there is no specific trend of INB from returning travelers alone with the change in quarantine length due to the trade-off that a longer quarantine results in higher productivity loss per traveler but fewer outgoing travelers.

### 3.3. Impacts of allowing unvaccinated individuals to enter Singapore under relaxed policy

All border control policies modeled effectively controlled COVID-19 transmission, keeping patient-days under the ICU capacity limit. The highest monthly number of ICU cases among travelers and secondary cases is 63.30 cases per month (Table 3), with 572.12 ICU patient-days, under the most relaxed policy of having no quarantine and no testing (S11 in Table 1). The unvaccinated traveler group causes fewer total secondary cases in the community compared to the vaccinated traveler group, as per the difference in the secondary case counts between the combined vaccinated and unvaccinated traveler group (Table 2) and the vaccinated only traveler group (Table 3). This is because there are fewer unvaccinated than vaccinated travelers, and unvaccinated travelers have a higher chance of being detected, especially through symptomatic infection. Given the small proportion of unvaccinated travelers, it would be acceptable to allow them entry into the country under the relaxed border control policy. This would also generate a higher total INB for Singapore.

### 3.4. Most and least cost-beneficial policies for Singapore

The most cost-beneficial (highest INB) and least cost-beneficial (lowest INB) policies do not differ between the group of vaccinated travelers and the combined group of vaccinated and unvaccinated travelers (Table 4) as the proportion of vaccinated travelers predominates in the latter. Likewise, the most and least cost-beneficial policies for inbound and returning travelers combined resemble those for the inbound travelers alone, except that the lowest INB policy for the combined group has PCR pre-departure and upon arrival (S10 in Table 1), due to the cost of testing returning travelers.

**Table 4:** Most and least cost-beneficial policies for different traveler groups and the resulting monthly sINB.

	**Most cost-beneficial policy**				**Least cost-beneficial policy**			
	**Length of quarantine**	**Testing strategy**	**INB (in millions)**		**Length of quarantine**	**Testing strategy**	**INB (in millions)**	
			**Vaccinated**	**Vaccinated and unvaccinated combined**			**Vaccinated**	**Vaccinated and unvaccinated combined**
**Inbound travelers**	0	S5	$1,687.81	$2,219.05	14	S11	–$2.29	–$3.00
**Returning travelers**	0	S11	$25.80	$25.50	2	S6	–$147.05	–$189.98
**Inbound travelers and returning travelers combined**	0	S5	$1,707.23	$2,236.24	14	S10	–$0.10	–$0.13

The most and least cost-beneficial policies specified in the table apply to both vaccinated traveler group and the combined group of vaccinated and unvaccinated travelers, because the most and least cost-beneficial policies for the two groups are the same, regardless of inbound travelers, returning travelers or combining inbound and returning travelers.

S5, S6, S10 and S11 are as specified in Table 1.

The policy with the highest INB for inbound travelers is no quarantine with ART pre-departure and upon arrival (S5 in Table 1). The policy with the lowest INB for inbound travelers is a 14-day quarantine with no test (S11 in Table 1). For returning travelers, the policy with the highest INB is no quarantine and no test (S11 in Table 1), which minimizes the cost of quarantining and testing travelers. The policy with the lowest INB for returning travelers is a 2-day quarantine with PCR pre-departure, upon arrival and exit quarantine, and ART every day during quarantine (S6 in Table 1). Under the costliest testing strategy S6, the 2-day quarantine results in a considerable number of returning travelers which maximizes the cost of quarantining and testing the returning travelers.

INB, incremental net benefit, compared to pre-opening period.

The highest INB from the combined group of all travelers is $2,236.24m, with $1,687.81m from vaccinated inbound travelers, $531.24m from unvaccinated inbound travelers, $19.42m from vaccinated returning travelers, and –$2.23m from unvaccinated returning travelers. The lowest INB from the combined group of all travelers is –$0.13m. The INB from inbound travelers ranges from –$3.00m to $2,219.05m, while the INB from returning travelers ranges from –$189.98m to $25.50m. The composition of highest and lowest INBs confirmed that economic gains from border opening are mainly from inbound travelers, and that having unvaccinated travelers enter Singapore under the same relaxed quarantine and testing policy as vaccinated travelers increases the economic gains.

### 3.5. Cost and benefit drivers

**Figure 1:**
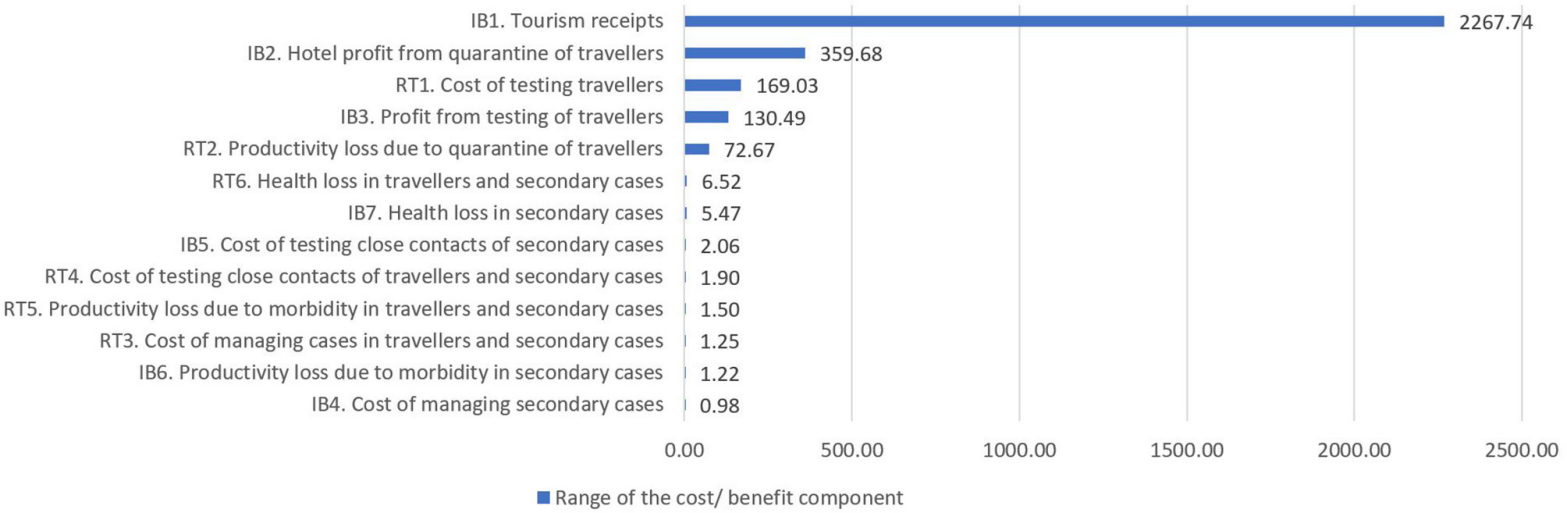
Range of cost/benefit components in the combined analysis (not vaccine differentiated). Cost/benefit components (corresponding to Supplementary Table A.7 in Supplementary material 3) are sorted from largest range to smallest range. Range is calculated as the difference between the highest and lowest instances of the 13 cost/benefit components across all policy options. The larger the range of a cost/benefit component, the more it contributes to driving which policy option has higher NMB or INB. IB, inbound traveler; RO, returning traveler; COVID-19, coronavirus disease 2019; NMB, net monetary benefit; INB, incremental net benefit, compared to pre-opening period.

Among the 13 cost/benefit components, the top driver for higher NMB or INB is the tourism receipts from inbound travelers, whose range is 6–2,321 times larger than the ranges of the other components (Figure 1). This finding confirms the economic importance of inbound tourism as the main contributor to economic gains from border opening. In general, the cost/benefit components related to tourism and border control policies have bigger economic impacts than components related to COVID-19 transmission. Among components related to COVID-19 transmission, the health losses of infected returning travelers and associated secondary cases have a larger economic impact than the costs of managing the COVID-19 cases.

### 3.6. Sensitivity analysis

The optimal policy in terms of highest total INB is robust to deviations of model assumptions from the base case (Table 5). The only exception is when the healthcare price level in destination countries is changed to the high estimate of 1.90 (Australia relative to Singapore), in which case no quarantine and no testing gives the highest INB. The highest total INB per month is generally robust. Exceptions include when the percentage reduction in the number of travelers per additional day of quarantine is halved, when the length of quarantine required to enter countries in the ROW is fixed at 0 or 14 days, or when the spill-over effects of increased tourism are incorporated in the calculation. The monthly total number of ICU cases (among travelers and secondary cases) under the optimal policy decreases noticeably when the length of entry quarantine in the ROW countries is fixed at 14 days. The monthly total number of ICU cases surges when the assumed vaccine coverage among inbound travelers is decreased to the rate of coverage with a full regimen plus a booster shot in the ROW as of June 2022, resulting in 92.04 ICU cases and 895.50 ICU patient-days per month under the most cost-beneficial policy, or when prevalence rates are at the highest observed rates from January to mid-June 2022 at 1.89% in Singapore and 0.28% in the ROW, resulting in 94.47 ICU cases and 851.48 ICU patient-days per month under the most cost-beneficial policy. There is no scenario in which the ICU capacity (3,000 patient-days) is exceeded.

**Table 5:** Deterministic sensitivity analysis for economically optimal policy.

**Varying parameter**	**Strategy with highest total INB**			
	**Testing strategy**	**Quarantine length (days)**	**Total INB (in millions)**	**Total ICU cases**
Base case	S5	0	$2,236.24	46.69
Percentage reduction in travelers with one more day of quarantine x0.5	S5	0	$1,632.84	46.69
Percentage reduction in travelers with one more day of quarantine x1.5	S5	0	$2,284.99	46.69
Quarantine length in ROW fixed at 0 days	S5	0	$1,692.95	46.69
Quarantine length in ROW fixed at 14 days	S5	0	$205.61	7.19
Prevalence as the minimum during January–June 2022	S5	0	$2,246.98	16.06
Prevalence as the maximum during January–June 2022	S5	0	$2,219.54	94.47
*R*_0_ = 10	S5	0	$2,233.57	50.78
*R*_0_ = 15	S5	0	$2,226.15	62.15
Vaccine coverage as the booster coverage in June 2022	S5	0	$2,225.93	92.04
Vaccine efficacy in ROW as the efficacy of inactivated vaccine	S5	0	$2,238.25	44.21
PCR turn around period of 2 days	S5	0	$2,236.23	46.69
Include spillover effect from tourism sector (multiplier = 2.37)	S5	0	$5,266.12	46.69
Productivity loss due to quarantine = 0%	S5	0	$2,205.02	46.69
Healthcare price ratio of ROW over Singapore as Indonesia over Singapore	S5	0	$2,237.79	46.69
Healthcare price ratio of ROW over Singapore as Australia over Singapore	S11	0	$2,231.31	63.30
Medical cost of COVID-19 cases x2	S5	0	$2,234.65	46.69
QALY high estimates	S5	0	$2,074.12	46.69
CET, 0.4 GDP per capita	S5	0	$2,240.35	46.69
CET, 3 GDP per capita	S5	0	$2,211.18	46.69

S5 and S11 are as specified in Table 1.

Total INB is the monthly INB from all the vaccinated and unvaccinated, inbound and returning travelers. Total ICU cases is the monthly total number of ICU cases among all travelers and their secondary cases.

INB, incremental net benefit, compared to pre-opening period; ICU, intensive care unit; ROW, rest of world; R_0_, basic reproduction number; COVID-19, coronavirus disease 2019; QALY, quality-adjusted life year; CET, cost-effectiveness threshold.

## 4. Discussion

### 4.1. Main findings

This case study aimed to understand the impacts of varying quarantine lengths and testing strategies at the Singapore border with both inbound travelers and returning travelers, and to quantify the epidemiological and economic impacts of allowing unvaccinated individuals to enter Singapore's borders under the same relaxed border-opening policies as vaccinated individuals. The economic impacts of testing and quarantine policies differed for inbound travelers and returning travelers. The biggest determinant for economic recovery was a shorter quarantine length, which led to a sharp increase in tourism receipts. Therefore, the optimal quarantine length is 0 days. With no quarantine, PCR entry test is not feasible due to the 1-day turnaround time assumption. However, no-testing strategy will result in higher international transmission and no profits from testing. Accordingly, a policy of no quarantine, with ART pre-departure and upon arrival (S5 in Table 1) stood out with the highest INB. Given the small proportion of unvaccinated travelers, allowing them to enter Singapore together with vaccinated travelers under the optimal policy will give Singapore a higher total INB, with ICU patient-day counts kept under the tolerance threshold. The optimal policy is robust under alternative model assumptions. The economically optimal policy is consistent with the recommendations of epidemiological studies on the use of pre-departure and on-arrival test (4, 36) with no or very short quarantine (37).

Our findings provide epidemiological transmission and economic evidence to support the selection of testing and quarantine policies for border reopening, with a focus on economic recovery whilst ensuring an acceptable number of ICU patient-days. Although the optimal policy identified in this study is based on total INB and total number of ICU cases, actual policy decision-making may rely on all columns in Tables 2, 3 concurrently.

Although studies recommended vaccination-status-dependent border control policies for efficient transmission control (38, 39), our findings suggest that we can afford to be less stringent about the vaccination status of travelers when the global vaccination rate reaches a certain threshold. When we consider the many challenges associated with developing an internationally recognized vaccination passport (40–42), the trade-offs of having imported cases through unvaccinated individuals appear to be acceptable. This is particularly so given that the validity of vaccination status is constantly evolving, being dependent on whether the vaccine is effective against emerging strains of the virus and whether the predominant strains of the virus are similar across different countries.

There is a paucity of data on the topic of border control strategies for infectious disease management, especially regarding the epidemiological-economic trade-offs to be made by a governing state. Border control policies have been found to reduce transmission from imported cases (3, 4, 36), but also impact the economy negatively, especially for the tourism sector (43, 44), although quarantine brings profit to accommodation industry (45). This study added to the strand of literature by modeling the trade-offs between various aspects of border control policy impacts and identifying the predominant impact in the Singapore setting.

Findings from this study will also help policymakers find an optimal border-control strategy that balances economic recovery with adequate disease control. While we used COVID-19 as an illustration, the calculations in this paper may be applied to any pandemic and the estimates can be revised by updating the model parameters with values that reflect the conditions of a future pandemic.

### 4.2. Limitations

Our study has several limitations. First, our analyses are valid in the context of stable prevalence, high vaccination rate, low ICU admission and accessibility to insurance which allow travelers to feel safe enough to travel. This means that the recommended policy options may not be optimal during the early phase of a pandemic. However, it remains that when vaccination coverage is sufficiently high (similar to levels assumed in this analysis), border control can be safely relaxed. Second, we did not include the intangible gains from overseas travel, including the downstream positive benefits on work productivity upon return from vacation and emotional gains from visiting family members. These are particularly pertinent in Singapore since more than one-third of the population are permanent residents or work pass holders with families living overseas. Third, other policy considerations in actuality may include capacity for PCR testing and the discomfort and inconvenience associated with PCR testing. However, these considerations support the optimal policy recommendation, in which our result suggests an optimal policy combination of ART without quarantine over any PCR-combination policies. Fourth, additional cases beyond the direct secondary cases resulting from imported infections are not accounted for. Thus, we may have underestimated transmission-related costs due to the further cases that would result from the imported infections, and hence overestimated the NMB. Fifth, the effect of border control policy on willingness to travel was simplified as a constant reduction rate in the number of travelers associated with one additional day of quarantine. A more careful econometric analysis could be conducted if data was available for different bilateral border control policies implemented between various countries and the resulting changes in the number of travelers under each policy.

### 4.3. Conclusions

The model we developed in this case study provides a comparison of costs and benefits in both health and economic terms, of different border control measures. This model can be applied to other countries with varying disease contexts to provide evidence for policy decisions in future pandemics. In this case study of Singapore in the context of the COVID-19 pandemic, relaxing border control to encourage tourism will increase the economic growth of Singapore, the destination country, even after taking into consideration the effects on local case load. Our model has shown that, given the current vaccination coverage in the world, incoming travelers, regardless of vaccination status, do not necessarily contribute to a high COVID case load and thus the economic value generated by relaxing border control will more than offset the associated economic costs. This should provide reassurance to countries that are striving for a stable situation with endemic COVID and for equity between vaccinated and unvaccinated travelers.

## Data availability statement

The original contributions presented in the study are included in the article/Supplementary material, further inquiries can be directed to the corresponding author.

## Author contributions

JL, NL, VH, and H-LW contributed to the conception and design of the study. CC, JL, NL, and H-LW acquired, analyzed and interpreted the data. JL, CC, and NL drafted the manuscript. BD, VH, and H-LW critically revised the manuscript. All authors participated sufficiently in the work to take public responsibility for appropriate portions of the content, gave final approval of the version to be published, and agreed to be accountable for all aspects of the work in ensuring that questions related to the accuracy or integrity of any part of the work are appropriately investigated and resolved.
