# Supplementary material for: Cost benefit analysis of alternative testing and quarantine policies for travelers for infection control: A case study of Singapore during the COVID-19 pandemic
Source: Front Public Health. 2023 Feb 23;11:1101986. doi: 10.3389/fpubh.2023.1101986 (PMC9996245; doi:10.3389/fpubh.2023.1101986)
Supplement: Supplementary file 1 [file Data_Sheet_1.docx]

Supplementary Material 1. Input value and data source of parameters

# Data Sheets

**Table A.1 Parameters on economics and tourism**

| **Parameter description** | | **Value** | **Data source** |
| --- | --- | --- | --- |
| Proportion of business travellers among all travellers | | 10% | Estimated based on yearly data from Singapore Tourism Board (STB) (Singapore Tourism Analytics Network, 2022) |
| Per capita expenditure of business travellers to Singapore | | $2305 |  |
| Per capita expenditure of tourists to Singapore | | $1408 |  |
| Monthly income of population in Singapore | | $4680 | Ministry of Manpower (MOM), Singapore (Ministry of Manpower Singapore, 2022) |
| Tourism multiplier in Singapore, only in deterministic sensitivity analysis (DSA) | | 2.37 | Estimated based on input-output table and tourism receipt component in Singapore (Department of Statistics, 2022; Singapore Tourism Analytics Network, 2022) |
| Cost effectiveness threshold (CET) of Singapore | base case | $75000 | Viswambaram et al. (Viswambaram, Wee, & LIM, 2020) |
|  | DSA #1 | $39119 | Supply-side CET of Singapore, assuming the ratio of Singapore’s CET over its GDP per capita is 0.4, same as the ratio of UK’s supply side CET over UK’s GDP per capita (Department of Statistics, 2022; Ochalek, Lomas, & Claxton, 2015) |
|  | DSA #2 | $293394 | Assume the CET of Singapore is 3 times of Singapore GDP per capita in 2021 (Department of Statistics, 2022; Marseille, Larson, Kazi, Kahn, & Rosen, 2014; Thokala, Ochalek, Leech, & Tong, 2018) |
| Ratio of healthcare cost in the destination countries for Singaporean outbound travellers relative to that in Singapore | base case | 0.3578 | Estimated by taking ratio of the weighted average of health expenditure per capita (HEPC) of Malaysia, Thailand, China, Indonesia, Australia, Japan and South Korea (major destination countries of Singaporean travellers) over the HEPC of Singapore in 2019 (World Health Organization, 2022a). The weights were estimated by number of Singaporean travellers to each destination country in 2019 (SINGSTAT, 2022). |
|  | DSA #1 | 0.0421 | Low estimates. Ratio of the HEPC of Indonesia over the HEPC of Singapore in 2019. |
|  | DSA #2 | 1.9002 | High estimates. Ratio of the HEPC of Australia over the HEPC of Singapore in 2019. |

**Table A.2 Parameters on border opening measures**

| **Parameter description** | | **Value** | **Data source** |
| --- | --- | --- | --- |
| Daily price of quarantine at Singapore | | $200 | Estimated based on “Stay Home Notice” hotel prices listed on Agoda |
| Percentage productivity loss if quarantined | base case | 30% | (van Ballegooijen, Goossens, Bruin, Michels, & Krol, 2021) |
|  | DSA | 0% | Assumed |
| Antigen sensitivity by log-viral load, logit constant coefficient | | -3.748627 | (Peto et al., 2021) |
| Antigen sensitivity by log-viral load, logit linear coefficient | | 1.080585 |  |
| PCR sensitivity by log-viral load, logit constant coefficient | | -2.67331 | (Miller et al., 2020) |
| PCR sensitivity by log-viral load, logit linear coefficient | | 0.929224 |  |
| Price of ART pre-departure test | | $25 | Local healthcare providers (HCPs). Assume remote supervision only for returning outbound travellers. |
| Price of ART entry test | | $15 | Ministry of Health (MOH), Singapore |
| Price of ART quarantine test | | $13 | Immigration & Checkpoints Authority (ICA), Singapore |
| Price of ART exit test | | $15 | Assumed to be equal to entry test |
| Price of PCR pre-departure test | | $150 | Local HCPs |
| Price of PCR entry test | | $160 | ICA Singapore |
| Price of PCR quarantine test | | $125 | Assumed to be equal to exit test |
| Price of PCR exit test | | $125 | ICA Singapore |
| Cost of ART test in Singapore | | $5 | Estimated based on advice by MOH, Singapore |
| Cost of PCR pre-departure test and confirmation test in Singapore | | $75 |  |
| Cost of PCR test upon arrival at Singapore | | $80 |  |
| Cost of PCR test during and exit quarantine in Singapore | | $63 |  |

**Table A.3 Risk profile of COVID-19 patients and vaccine efficacy**

| **Parameter description** | | | **Value** | **Data source** |
| --- | --- | --- | --- | --- |
| Unvaccinated secondary cases in the Singapore community | Probability of asymptomatic | | 30.00% | Estimated based on an age-specific risk profile (Menni et al., 2022; O’Driscoll et al., 2021; Oran & Topol, 2020; Sheikh, McMenamin, Taylor, & Robertson, 2021; Wei et al., 2020)  and different age structures of Singapore local population and travellers (Department of Statistics, 2022; Singapore Tourism Analytics Network, 2022) |
|  | Probability of mild/ moderate symptoms | | 59.02% |  |
|  | Probability of hospitalization | | 7.17% |  |
|  | Probability of ICU | | 1.55% |  |
|  | Probability of death | | 0.67% |  |
| Unvaccinated cases among travellers | Probability of asymptomatic | | 30.00% |  |
|  | Probability of mild/ moderate symptoms | | 61.51% |  |
|  | Probability of hospitalization | | 5.69% |  |
|  | Probability of ICU | | 1.24% |  |
|  | Probability of death | | 0.32% |  |
| Vaccine in Singapore | Coverage in general population | base case | 92% | MOH, Singapore (Ministry of Health Singapore, 2022b)  The base case value is the coverage of 2-3 doses as of June 2021, and DSA value is the coverage of 3 doses as of June 2021 |
|  |  | DSA | 78% |  |
|  | Percentage reduction against infection | | 61% | Estimated based on efficacy of 2-3 doses mRNA vaccine (Chemaitelly et al., 2022; Tseng et al., 2022) and coverage of 2 doses and 3 doses. |
|  | Percentage reduction against symptomatic infection | | 45% |  |
|  | Percentage reduction against hospitalization and death | | 97% |  |
| Vaccine in the ROW | Coverage in general population | base case | 76% | A weighted average of country-specific vaccine coverage (World Health Organization, 2022b), with weights proportional to population size (World Bank, 2022).  The base case value is the coverage of 2-3 doses as of June 2021, and DSA value is the coverage of 3 doses as of June 2021. |
|  |  | DSA | 30% |  |
|  | Percentage reduction against infection | base case | 36% | Estimated based on efficacy of 2-3 doses mRNA vaccine (Chemaitelly et al., 2022; Tseng et al., 2022) for base case or 2 doses inactivated vaccine (McMenamin et al., 2022) for DSA and coverage of 2 doses and 3 doses. |
|  |  | DSA | 18% |  |
|  | Percentage reduction against symptomatic infection | base case | 45% |  |
|  |  | DSA | 18% |  |
|  | Percentage reduction against hospitalization and death | base case | 90% |  |
|  |  | DSA | 83% |  |

**Table A.4 Parameters on cost related to COVID-19 cases**

| **Parameter description** | | **Value** | **Data source** |
| --- | --- | --- | --- |
| Cost of treating an unvaccinated case in Singapore | asymptomatic | $42.50 | Estimated based on data shared by MOH and NCID, Singapore, taking into account cost of hospitalization, tests, and transport. |
|  | with mild/moderate symptoms | $67.50 |  |
|  | hospitalized | $10640 |  |
| Cost of treating a vaccinated case in Singapore | asymptomatic | $25.00 |  |
|  | with mild/moderate symptoms | $38.75 |  |
|  | hospitalized | $7642.98 |  |
| Number of days isolated at home | unvaccinated asymptomatic or with mild/moderate symptoms | 8.5 | Estimated based on COVID management practice by MOH in June 2022 (Ministry of Health Singapore, 2022c) |
|  | vaccinated asymptomatic or with mild/moderate symptoms | 5 |  |
| Number of days in general ward | unvaccinated hospitalized but non-ICU cases | 20 | Estimated based on data from MOH and NCID, Singapore |
|  | unvaccinated ICU cases | 11 |  |
|  | vaccinated hospitalized but non-ICU cases | 14 |  |
|  | vaccinated ICU cases | 15 |  |
| Number of days in ICU | unvaccinated | 10 |  |
|  | vaccinated | 6 |  |
| Length of medical leave if seeing a GP (days) | | 0.5 | Assumed |
| Percentage productivity loss if hospitalized | | 100% | Assumed |
| Percentage productivity loss if a case is not hospitalized (asymptomatic/mild/moderate cases) | | 30% | Assumed to be the same as productivity loss due to quarantine. |
| Number of close contacts per case | | 16.76 | Estimated based on data from MOH, Singapore |
| Cost of testing close contacts of one more case in Singapore | | $419.00 | Estimated based on COVID management practice by MOH in June 2022 (Ministry of Health Singapore, 2022d), with assumption of 5 ARTs per close contact |

**Table A.5 Parameters on health outcome**

| **Parameter description** | | | **Value** | **Data source** |
| --- | --- | --- | --- | --- |
| Health-related quality of life (HRQoL) of Singapore general population | | | 0.95 | (Abdin, Subramaniam, Vaingankar, Luo, & Chong, 2015) |
| Percentage reduction in HRQoL due to isolation | | | 2.92% | (Wong et al., 2020) |
| Quality-adjusted life years (QALY) loss of an unvaccinated case | asymptomatic | | 0.000646 | In base cases analysis, QALY loss due to morbidity were estimated based on HRQoL scores of COVID patients and isolated people, length of isolation and hospitalization, length and probability of long COVID (Al-Aly, Bowe, & Xie, 2022; Poteet & Craig, 2021; Poudel et al., 2021; Wong et al., 2020). QALY loss due to mortality were estimated based on average age of COVID-19 mortality and life expectancy of Singapore general population, discounted by 3%.  The QALY loss due to morbidity in DSA for symptomatic cases were high estimates from literature based on other respiratory disease (Basu & Gandhay, 2021), with an assumption that vaccination saves 10% of QALY loss from COVID symptom. |
|  | with mild/ moderate symptoms | base case | 0.003480 |  |
|  |  | DSA | 0.43 |  |
|  | hospitalized | base case | 0.162256 |  |
|  |  | DSA | 0.523564 |  |
|  | deceased | base case | 8.777678 |  |
|  |  | DSA | 9.138985 |  |
| QALY loss of a vaccinated case | asymptomatic | | 0.000380 |  |
|  | with mild/ moderate symptoms | base case | 0.002047 |  |
|  |  | DSA | 0.387 |  |
|  | hospitalized | base case | 0.136652 |  |
|  |  | DSA | 0.471207 |  |
|  | deceased | base case | 8.752074 |  |
|  |  | DSA | 9.086629 |  |

**Table A.6 Parameters on COVID-19 transmission**

| **Parameter description** | | **Value** | **Data source** |
| --- | --- | --- | --- |
| Reproduction rate (R0) without effect of vaccines | base case | 8.2 | Liu et al. (Liu & Rocklöv, 2022) |
|  | DSA #1 | 10 | Butki (Burki, 2022) |
|  | DSA #2 | 15 | Hypothetical high estimates |
| Prevalence in Singapore | base case | 0.0091794 | Derived based on daily case counts from WHO COVID dashboard (World Health Organization, 2022b), population size (World Bank, 2022), and eight-day disease duration with Omicron on average (Menni et al., 2022).  The ROW excluded countries with missing case counts or population size or in Africa.  Base case value is the mean in 2022, with min and max in DSA. |
|  | DSA #1 | 0.0032778 |  |
|  | DSA #2 | 0.018994 |  |
| Prevalence in the ROW | base case | 0.0013669 |  |
|  | DSA #1 | 0.0004705 |  |
|  | DSA #2 | 0.0027718 |  |
| Scale-up factor for prevalence among travellers compared to general population | | 2.63 | Calibrated based on number of travellers and imported cases in May 2022 (Ministry of Health Singapore, 2022a; Singapore Tourism Analytics Network, 2022; Yusof, 2022) |
| Inbound travellers’ length of stay in Singapore (days) | | 3.42 | STB (Singapore Tourism Analytics Network, 2022) |
| Outbound travellers’ length of stay in the destination country (days) | | 7 | Assumed |
| Mean initial log-viral load | | 1.0084 |  |
| Mean slope of increasing log-viral load | | 2.837185387 | (Singanayagam et al., 2022; Young et al., 2022) |
| Mean slope of decreasing log-viral load | | -0.95 |  |
| Mean peak log-viral load | | 7.6584 |  |
| Mean days from peak log-viral load to symptoms | | 0.6 |  |

# References

Abdin, E., Subramaniam, M., Vaingankar, J. A., Luo, N., & Chong, S. A. (2015). Population norms for the EQ-5D index scores using Singapore preference weights. Quality of Life Research, 24(6), 1545–1553. https://doi.org/10.1007/s11136-014-0859-5

Al-Aly, Z., Bowe, B., & Xie, Y. (2022). Long COVID after breakthrough SARS-CoV-2 infection. Nature Medicine, 1–7. https://doi.org/10.1038/s41591-022-01840-0

Basu, A., & Gandhay, V. J. (2021). Quality-Adjusted Life-Year Losses Averted With Every COVID-19 Infection Prevented in the United States. Value in Health, 24(5), 632–640. https://doi.org/10.1016/j.jval.2020.11.013

Burki, T. K. (2022). Omicron variant and booster COVID-19 vaccines. The Lancet Respiratory Medicine, 10(2), e17. https://doi.org/10.1016/S2213-2600(21)00559-2

Chemaitelly, H., Ayoub, H. H., AlMukdad, S., Coyle, P., Tang, P., Yassine, H. M., … Al-Kanaani, Z. (2022). Duration of mRNA vaccine protection against SARS-CoV-2 Omicron BA. 1 and BA. 2 subvariants in Qatar. Nature Communications, 13(1), 1–12. https://doi.org/10.1101/2022.03.13.22272308

Department of Statistics. (2022). National Accounts. Retrieved from https://www.singstat.gov.sg/find-data/search-by-theme/economy/national-accounts/publications-and-methodology

Liu, Y., & Rocklöv, J. (2022). The effective reproductive number of the Omicron variant of SARS-CoV-2 is several times relative to Delta. Journal of Travel Medicine, 29(3), taac037. https://doi.org/10.1093/jtm/taac037

Marseille, E., Larson, B., Kazi, D. S., Kahn, J. G., & Rosen, S. (2014). Thresholds for the cost–effectiveness of interventions: alternative approaches. Bulletin of the World Health Organization, 93, 118–124. https://doi.org/10.2471/BLT.14.138206

McMenamin, M. E., Nealon, J., Lin, Y., Wong, J. Y., Cheung, J. K., Lau, E. H. Y., … Cowling, B. J. (2022). Vaccine effectiveness of two and three doses of BNT162b2 and CoronaVac against COVID-19 in Hong Kong. MedRxiv. https://doi.org/10.1101/2022.03.22.22272769

Menni, C., Valdes, A. M., Polidori, L., Antonelli, M., Penamakuri, S., Nogal, A., … Hu, C. (2022). Symptom prevalence, duration, and risk of hospital admission in individuals infected with SARS-CoV-2 during periods of omicron and delta variant dominance: a prospective observational study from the ZOE COVID Study. The Lancet, 399(10335), 1618–1624. https://doi.org/10.1016/S0140-6736(22)00327-0

Miller, T. E., Garcia Beltran, W. F., Bard, A. Z., Gogakos, T., Anahtar, M. N., Astudillo, M. G., … Mahowald, G. K. (2020). Clinical sensitivity and interpretation of PCR and serological COVID‐19 diagnostics for patients presenting to the hospital. The FASEB Journal, 34(10), 13877–13884. https://doi.org/10.1096/fj.202001700RR

Ministry of Health Singapore. (2022a). Data on COVID-19 cases. Retrieved June 20, 2022, from https://data.gov.sg/dataset/covid-19-case-numbers?resource_id=400a3eb4-8702-4050-9700-988bfea7a20f

Ministry of Health Singapore. (2022b). VACCINATION STATISTICS. Retrieved June 20, 2022, from https://www.moh.gov.sg/covid-19/vaccination/statistics

Ministry of Health Singapore. (2022c). Well and Test Positive, or Condition Assessed Mild by Doctor. Retrieved June 20, 2022, from https://www.covid.gov.sg/well-and-positive-or-condition-assessed-mild-by-doctor

Ministry of Health Singapore. (2022d). What To Do Next? Retrieved June 20, 2022, from https://www.covid.gov.sg/exposed/filter

Ministry of Manpower Singapore. (2022). Summary Table: Income. Retrieved June 15, 2022, from https://stats.mom.gov.sg/Pages/Income-Summary-Table.aspx

O’Driscoll, M., Dos Santos, G. R., Wang, L., Cummings, D. A. T., Azman, A. S., Paireau, J., … Salje, H. (2021). Age-specific mortality and immunity patterns of SARS-CoV-2. Nature, 590(7844), 140–145. https://doi.org/10.1038/s41586-020-2918-0

Ochalek, J. M., Lomas, J., & Claxton, K. P. (2015). Cost per DALY averted thresholds for low-and middle-income countries: evidence from cross country data. Retrieved from http://www.york.ac.uk/media/che/documents/papers/researchpapers/CHERP122_cost_DALY_LMIC_threshold.pdf

Oran, D. P., & Topol, E. J. (2020). Prevalence of asymptomatic SARS-CoV-2 infection: a narrative review. Annals of Internal Medicine, 173(5), 362–367. https://doi.org/10.7326/M20-3012

Peto, T., Affron, D., Afrough, B., Agasu, A., Ainsworth, M., Allanson, A., … Ashbridge, N. (2021). COVID-19: Rapid antigen detection for SARS-CoV-2 by lateral flow assay: A national systematic evaluation of sensitivity and specificity for mass-testing. EClinicalMedicine, 36, 100924. https://doi.org/https://doi.org/10.1016/j.eclinm.2021.100924

Poteet, S., & Craig, B. M. (2021). QALYs for COVID-19: a comparison of US EQ-5D-5L value sets. The Patient-Patient-Centered Outcomes Research, 14(3), 339–345. https://doi.org/10.1007/s40271-021-00509-z

Poudel, A. N., Zhu, S., Cooper, N., Roderick, P., Alwan, N., Tarrant, C., … Yao, G. L. (2021). Impact of Covid-19 on health-related quality of life of patients: A structured review. PLoS One, 16(10), e0259164. https://doi.org/10.1371/journal.pone.0259164

Sheikh, A., McMenamin, J., Taylor, B., & Robertson, C. (2021). SARS-CoV-2 Delta VOC in Scotland: demographics, risk of hospital admission, and vaccine effectiveness. The Lancet. https://doi.org/10.1016/S0140-6736(21)01358-1

Singanayagam, A., Hakki, S., Dunning, J., Madon, K. J., Crone, M. A., Koycheva, A., … Varro, R. (2022). Community transmission and viral load kinetics of the SARS-CoV-2 delta (B. 1.617. 2) variant in vaccinated and unvaccinated individuals in the UK: a prospective, longitudinal, cohort study. The Lancet Infectious Diseases, 22(2), 183–195. https://doi.org/10.1016/S1473-3099(21)00648-4

Singapore Tourism Analytics Network. (2022). Singapore Tourism Analytics Network. Retrieved June 15, 2022, from Singapore Tourism Board website: http://www.singstat.gov.sg/find-data/search-by-theme/economy/national-accounts/publications-and-methodology

SINGSTAT. (2022). General Household Survey, Release 2: Overseas Travel. Retrieved June 15, 2022, from SINGSTAT website: https://www.singstat.gov.sg/-/media/files/publications/ghs/general_household_survey_release2/chap2.pdf

Thokala, P., Ochalek, J., Leech, A. A., & Tong, T. (2018). Cost-effectiveness thresholds: the past, the present and the future. Pharmacoeconomics, 36(5), 509–522. https://doi.org/10.1007/s40273-017-0606-1

Tseng, H. F., Ackerson, B. K., Luo, Y., Sy, L. S., Talarico, C. A., Tian, Y., … Ku, J. H. (2022). Effectiveness of mRNA-1273 against SARS-CoV-2 Omicron and Delta variants. Nature Medicine, 28(5), 1063–1071. https://doi.org/10.1038/s41591-022-01753-y

van Ballegooijen, H., Goossens, L., Bruin, R. H., Michels, R., & Krol, M. (2021). Concerns, quality of life, access to care and productivity of the general population during the first 8 weeks of the coronavirus lockdown in Belgium and the Netherlands. BMC Health Services Research, 21(1), 227. https://doi.org/10.1186/s12913-021-06240-7

Viswambaram, A., Wee, Y. R., & LIM, S. (2020). Is There an Implicit Willingness-to-Pay Threshold in Singapore? Value in Health Regional Issues, 22, S72. Retrieved from https://www.valuehealthregionalissues.com/article/S2212-1099(20)30427-1/pdf

Wei, E. W., Heng, T. C., Chan, M., TOng, T. T., Pada, S. K., Archuleta, S., … Cheng, T. K. (2020). Age and chest radiography as possible parameters for rapid triage in COVID-19 outbreak surge. https://doi.org/10.21203/rs.3.rs-88104/v1

Wong, E. L.-Y., Ho, K.-F., Wong, S. Y.-S., Cheung, A. W.-L., Yau, P. S.-Y., Dong, D., & Yeoh, E.-K. (2020). Views on workplace policies and its impact on health-related quality of life during coronavirus disease (COVID-19) pandemic: cross-sectional survey of employees. International Journal of Health Policy and Management. https://doi.org/10.34172/ijhpm.2020.127

World Bank. (2022). Population, total. Retrieved June 19, 2022, from World Bank website: https://data.worldbank.org/indicator/SP.POP.TOTL

World Health Organization. (2022a). Global Health Expenditure Database. Retrieved June 15, 2022, from World Health Organization website: https://apps.who.int/nha/database/Select/Indicators/en

World Health Organization. (2022b). WHO Coronavirus (COVID-19) Dashboard. Retrieved June 13, 2022, from https://covid19.who.int/data

Young, B., Fong, S.-W., Chang, Z. W., Tan, K. Sen, Rouers, A., Goh, Y. S., … Chua, S. L. (2022). Comparison of the clinical features, viral shedding and immune response in vaccine breakthrough infection by the Omicron and Delta variants. https://doi.org/10.21203/rs.3.rs-1281925/v1

Yusof, A. (2022). More than 165,000 daily crossings at Singapore-Malaysia land borders since Apr 1: Johor government. Retrieved June 20, 2022, from Channel News Asia website: https://www.channelnewsasia.com/asia/singapore-johor-land-crossing-165000-daily-reopening-onn-hafiz-2758021
