# Supplementary material for: Cost benefit analysis of alternative testing and quarantine policies for travelers for infection control: A case study of Singapore during the COVID-19 pandemic
Source: Front Public Health. 2023 Feb 23;11:1101986. doi: 10.3389/fpubh.2023.1101986 (PMC9996245; doi:10.3389/fpubh.2023.1101986)
Supplement: Supplementary file 2 [file Data_Sheet_2.docx]

Supplementary Material 2. Additional details about transmission model

# Description

The inbound travellers were initialized with uniformly distributed days of infection (from 1 to 28 days prior to travel), with prevalence rate of 0.49% among unvaccinated travellers and 0.32% among vaccinated travellers. The prevalence rate were estimated based on the average prevalence rate (0.13%) in the rest of world (ROW) during Jan-Jun 2022 (World Health Organization, 2022), vaccination coverage and efficacy in the ROW (World Health Organization, 2022)(Tseng et al., 2022)(Chemaitelly et al., 2022), and a multiplier 2.63 to capture inflated infections due to over-activeness of travellers compared to general population. The multiplier was calibrated based on actual number of imported cases in May 2022 (Ministry of Health Singapore, 2022)(Yusof, 2022).

The returning outbound travellers were initialized with the vaccination-status-stratified prevalence in Singapore, followed by having days of infection that are geometrically distributed (from 1 to 7 days, as the length of stay in the community overseas is assumed to be 7 days), taking into account vaccination efficacy and coverage as well as over-activeness of travellers. Before outbound travellers return to Singapore, the estimated prevalence rate was 0.59% among the unvaccinated group and 0.15% among the vaccinated group.

Test sensitivities were computed based on average viral loads by day of infection, using estimated sensitivities by viral load of PCR (Miller et al., 2020) and ART (Peto et al., 2021). The incubation period was assumed to be log-normally distributed with mean 1.434 and standard deviation 0.661, and the infectious period is assumed to be normally distributed with mean 10 and standard deviation 1.5.(Singanayagam et al., 2022) Transmissions caused by each traveller are computed as the ratio of the number of days spent in Singapore (no upper limit for returning travellers, maximum 7 days for visiting travellers) while infected (post-incubation period) to 10 (the mean infectious period), multiplied by the assumed value of R0 for Omicron.

# References

Chemaitelly, H., Ayoub, H. H., AlMukdad, S., Coyle, P., Tang, P., Yassine, H. M., … Al-Kanaani, Z. (2022). Duration of mRNA vaccine protection against SARS-CoV-2 Omicron BA. 1 and BA. 2 subvariants in Qatar. Nature Communications, 13(1), 1–12. https://doi.org/10.1101/2022.03.13.22272308

Miller, T. E., Garcia Beltran, W. F., Bard, A. Z., Gogakos, T., Anahtar, M. N., Astudillo, M. G., … Mahowald, G. K. (2020). Clinical sensitivity and interpretation of PCR and serological COVID‐19 diagnostics for patients presenting to the hospital. The FASEB Journal, 34(10), 13877–13884. https://doi.org/10.1096/fj.202001700RR

Ministry of Health Singapore. (2022). Data on COVID-19 cases. Retrieved June 20, 2022, from https://data.gov.sg/dataset/covid-19-case-numbers?resource_id=400a3eb4-8702-4050-9700-988bfea7a20f

Peto, T., Affron, D., Afrough, B., Agasu, A., Ainsworth, M., Allanson, A., … Ashbridge, N. (2021). COVID-19: Rapid antigen detection for SARS-CoV-2 by lateral flow assay: A national systematic evaluation of sensitivity and specificity for mass-testing. EClinicalMedicine, 36, 100924. https://doi.org/10.1016/j.eclinm.2021.100924

Singanayagam, A., Hakki, S., Dunning, J., Madon, K. J., Crone, M. A., Koycheva, A., … Varro, R. (2022). Community transmission and viral load kinetics of the SARS-CoV-2 delta (B. 1.617. 2) variant in vaccinated and unvaccinated individuals in the UK: a prospective, longitudinal, cohort study. The Lancet Infectious Diseases, 22(2), 183–195. https://doi.org/10.1016/S1473-3099(21)00648-4

Tseng, H. F., Ackerson, B. K., Luo, Y., Sy, L. S., Talarico, C. A., Tian, Y., … Ku, J. H. (2022). Effectiveness of mRNA-1273 against SARS-CoV-2 Omicron and Delta variants. Nature Medicine, 28(5), 1063–1071. https://doi.org/10.1038/s41591-022-01753-y

World Health Organization. (2022). WHO Coronavirus (COVID-19) Dashboard. Retrieved June 13, 2022, from https://covid19.who.int/data

Yusof, A. (2022). More than 165,000 daily crossings at Singapore-Malaysia land borders since Apr 1: Johor government. Retrieved June 20, 2022, from Channel News Asia website: https://www.channelnewsasia.com/asia/singapore-johor-land-crossing-165000-daily-reopening-onn-hafiz-2758021
