# Supplementary material for: Cost benefit analysis of alternative testing and quarantine policies for travelers for infection control: A case study of Singapore during the COVID-19 pandemic
Source: Front Public Health. 2023 Feb 23;11:1101986. doi: 10.3389/fpubh.2023.1101986 (PMC9996245; doi:10.3389/fpubh.2023.1101986)
Supplement: Supplementary file 3 [file Data_Sheet_3.docx]

Supplementary Material 3. Additional details about economic quantification

# Data Sheets

**Table A.7 Net monetary benefit (NMB) components from inbound travellers and returning travellers**

| **Category** | **NMB components from inbound travellers (IB)** | **NMB components from returning travellers (RT)** |
| --- | --- | --- |
| Cost/benefit related to tourism and border control policies | IB1. (+) Tourism receipts from inbound travellers  IB2. (+) Hotel profit from quarantining inbound travellers  IB3. (+) Profit from testing inbound travellers | RT1. (-) Cost of testing returning outbound travellers  RT2. (-) Productivity loss due to quarantining returning outbound travellers |
| Cost due to COVID-19 | IB4. (-) Cost of managing secondary cases caused by inbound travellers  IB5. (-) Cost of testing close contacts of secondary cases caused by inbound travellers  IB6. (-) Productivity loss due to COVID-19 morbidity in secondary cases caused by inbound travellers | RT3. (-) Cost of managing cases in returning outbound travellers and related secondary cases   - Cost of managing outbound travellers diagnosed by pre-departure test - (-) Cost of managing returning outbound travellers diagnosed on arrival or during quarantine - (-) Cost of managing returning cases missed on arrival or during quarantine but develop symptoms afterwards - (-) Cost of managing secondary cases caused by returning outbound travellers who are not detected on arrival or during quarantine - RT4. (-) Cost of testing close contacts of returning outbound travellers and related secondary cases - RT5. (-) Productivity loss due to COVID-19 morbidity in returning outbound travellers and related secondary cases |
| Health loss due to COVID-19 | IB7. (-) Health loss due to COVID-19 morbidity and mortality in secondary cases caused by inbound travellers | RT6. (-) Health loss due to COVID-19 morbidity and mortality in returning travellers and related secondary cases |

# Calculation Details

## Variables from transmission model

- Unvaccinated inbound travellers

${d0}_{BA}^{UV}$ is number of unvaccinated cases who intend to travel to Singapore, but stopped by pre-test at their source country border

${d1}_{BA}^{UV}$ is number of unvaccinated cases travelling to Singapore, diagnosed at Singapore border

${ud1}_{BA}^{UV}$ is number of unvaccinated cases travelling to Singapore, missed by Singapore fence

${m1}_{BA}^{UV}$ is number of secondary cases, caused by unvaccinated cases travelling to Singapore and missed by Singapore fence

${Puds}_{BA}^{UV}$ is the probability that an unvaccinated inbound case missed by quarantine and testing later becomes symptomatic

- Vaccinated inbound travellers

${d0}_{BA}^{V}$ is number of vaccinated cases who intend to travel to Singapore, but stopped by pre-test at their source country fence

${d1}_{BA}^{V}$ is number of vaccinated cases travelling to Singapore, diagnosed at Singapore fence

${ud1}_{BA}^{V}$ is number of vaccinated cases travelling to Singapore, missed by Singapore fence

${m1}_{BA}^{V}$ is number of secondary cases, caused by vaccinated cases travelling to Singapore and missed by Singapore fence

${Puds}_{BA}^{V}$ is the probability that a vaccinated inbound case missed by quarantine and testing later becomes symptomatic

- Unvaccinated returning travellers

${d00}_{AB}^{UV}$ is number of unvaccinated cases who are diagnosed by pre-test at other countries’ fence before they go back to Singapore

${d2}_{AB}^{UV}$ is number of unvaccinated cases returning to Singapore, diagnosed at Singapore fence

${ud2}_{AB}^{UV}$ is number of unvaccinated cases returning to Singapore, missed by Singapore fence

${m2}_{AB}^{UV}$ is number of secondary cases, caused by unvaccinated cases returning to Singapore and missed by Singapore fence

${Puds}_{AB}^{UV}$ is the probability that an unvaccinated returning case missed by quarantine and testing later becomes symptomatic

- Vaccinated returning travellers

${d00}_{AB}^{V}$ is number of vaccinated cases who are diagnosed by pre-test at other countries’ fence before they go back to Singapore

${d2}_{AB}^{V}$ is number of vaccinated cases returning to Singapore, diagnosed at Singapore fence

${ud2}_{AB}^{V}$ is number of vaccinated cases returning to Singapore, missed by Singapore fence

${m2}_{AB}^{V}$ is number of secondary cases, caused by vaccinated cases returning to Singapore and missed by Singapore fence

${Puds}_{AB}^{V}$ is the probability that a vaccinated returning case missed by quarantine and testing later becomes symptomatic

## Willingness to travel

- Number of potential inbound travellers

$$N_{BA}^{UV}=1533433\times(1-\chi_{B})\times{(1-14.49\%)}^{2\times q_{A}^{UV}}$$

$$N_{BA}^{V}=1533433\times\chi_{B}\times{(1-14.49\%)}^{2\times q_{A}^{V}}$$

- $\chi_{B}$ is the vaccine coverage in ROW
- Number of potential outbound travellers

$$N_{AB}^{UV}=857754\times(1-\chi_{A})\times{(1-9.66\%)}^{2\times q_{A}^{UV}}$$

$$N_{AB}^{V}=857754\times\chi_{A}\times{(1-9.66\%)}^{2\times q_{A}^{V}}$$

- $\chi_{A}$ is the vaccine coverage in Singapore
- DSA:
- DSA #1: percentage reduction with one more day of quarantine ($14.49\%$ and $9.66\%$) increase and dicreases by 50%
- DSA #2: change $2\times q_{A}$ into $q_{A}$ (assume ROW requires no quarantine) and $(7+q_{A}$) (assume ROW requires 7-day quarantine)
- DSA #3: change in vaccine coverage

## Net benefit: Inbound travellers

1. (+) Tourism receipts from inbound travellers

$$\left( N_{BA}^{UV}-{d0}_{BA}^{UV} \right)\times\left( 1-{Pbiz}_{BA} \right)\times\gamma_{BA}+\left( N_{BA}^{UV}-{d0}_{BA}^{UV} \right)\times{Pbiz}_{BA}\times\alpha_{BA}$$

$$+(N_{BA}^{V}-{d0}_{BA}^{V})\times\left( 1-{Pbiz}_{BA} \right)\times\gamma_{BA}+(N_{BA}^{V}-{d0}_{BA}^{V})\times{Pbiz}_{BA}\times\alpha_{BA}$$

- $\gamma_{BA}$ is per-capita expenditure of inbound tourists (including accommodation after quarantine, aviation, medical, shopping, F&B, sightseeing, etc.)
- $\alpha_{BA}$ is per-capita expenditure of business travellers, assumed to be twice of $\gamma_{BA}$
- ${Pbiz}_{BA}$ is the proportion of business travellers among all travellers coming to Singapore

1. (+) Profit from quarantining inbound travellers

$$[\left( N_{BA}^{UV}-{d0}_{BA}^{UV} \right)\times q_{A}^{UV}$$

$$+\left( N_{BA}^{V}-{d0}_{BA}^{V} \right)\times q_{A}^{V}]\times\rho_{A}$$

- $\rho_{A}$ is the daily price of quarantine at Singapore

1. (+) Profit from testing inbound travellers

$$\left[ \begin{aligned} n1ART\left( t_{BA}^{UV} \right)\times p1ART+ n2ART\left( t_{BA}^{UV} \right)\times p2ART+n3ART\left( t_{BA}^{UV} \right)\times p3ART \\ +n1PCR\left( t_{BA}^{UV} \right)\times p1PCR+ n2PCR\left( t_{BA}^{UV} \right)\times p2PCR+n3PCR\left( t_{BA}^{UV} \right)\times p3PCR \end{aligned} \right]\times N_{BA}^{UV}$$

$$\left[ \begin{aligned} n1ART\left( t_{BA}^{V} \right)\times p1ART+ n2ART\left( t_{BA}^{V} \right)\times p2ART+n3ART\left( t_{BA}^{V} \right)\times p3ART \\ +n1PCR\left( t_{BA}^{V} \right)\times p1PCR+ n2PCR\left( t_{BA}^{V} \right)\times p2PCR+n3PCR\left( t_{BA}^{V} \right)\times p3PCR \end{aligned} \right]\times N_{BA}^{V}$$

- $n1ART\left( t \right)$ is the number of ART entry test per traveller, with value 0 or 1
- $n2ART\left( t \right)$ is the number of ART quarantine test
- $n3ART\left( t \right)$ is the number of ART exit test, with value 0 or 1
- $n1PCR\left( t \right)$ is the number of PCR entry test per traveller, with value 0 or 1
- $n2PCR\left( t \right)$ is the number of PCR quarantine test
- $n3PCR\left( t \right)$ is the number of PCR exit test, with value 0 or 1
- $p1ART$ is the profit per ART entry test
- $p2ART$ is the profit per ART quarantine test
- $p3ART$ is the profit per ART exit test
- $p1PCR$ is the profit per PCR entry test
- $p2PCR$ is the profit per PCR quarantine test
- $p3PCR$ is the profit per PCR exit test

1. (-) Direct cost of managing secondary cases caused by infectious inbound travellers who are not detected on arrival or during quarantine

$$-({m1}_{BA}^{UV}+{m1}_{BA}^{V})\times\varepsilon_{AA}$$

- $\varepsilon_{AA}$ is the expected cost of treating a local/ secondary case infected by inbound travellers / returning travellers

$$\varepsilon_{AA}=\frac{1-\chi_{A}}{1-\chi_{A}\times\mu_{A}}\varepsilon\left( P_{A}^{UV},{TC}_{A}^{UV} \right)+\frac{\chi_{A}-\chi_{A}\times\mu_{A}}{1-\chi_{A}\times\mu_{A}}\varepsilon\left( P_{A}^{V},{TC}_{A}^{V} \right)$$

- $\mu_{A}$ is the vaccine efficacy against infection in Singapore, i.e. reduced chance against infection
- $\nu_{A}$ is the vaccine efficacy against symptomatic infection in Singapore, i.e. reduced chance against symptomatic infection
- $\upsilon_{A}$ is the vaccine efficacy against severe/ critical cases in Singapore, i.e. reduced chance against severe/ critical cases
- $\chi_{A}$ is the vaccine coverage in Singapore
- $\boldsymbol{\varepsilon}\left( \boldsymbol{P,}\boldsymbol{TC}_{\boldsymbol{K}} \right)$ is the expected cost of treating an case at country K, whose probabilities of being asymptomatic/ mild/ moderate/ severe/ critical/ dead are $P$.

$$\varepsilon\left( P,{TC}_{K} \right)$$

$$=\varepsilon\left( \left( Pasp,Pmm,Psc,Pdth \right),\left( {TCasp}_{K},{TCmm}_{K},{TCsc}_{K} \right) \right)$$

$$=Pasp\times{TCasp}_{K}+Pmm\times{TCmm}_{K}+(Psc+Pdth)\times{TCsc}_{K}$$

- $P=\left( Pasp,Pmm,Psc,Pdth \right)$ in which
  - $Pasp$ is the probability of being asymptomatic cases
  - $Pmm$ is the proportion of cases with mild/moderate symptoms
  - $Psc$ is the proportion of cases with severe/critical symptoms but survive
  - $Pdth$ is the death rate, assumed with severe/critical symptoms
  - Note that $Pasp+Pmm+Psc+Pdth=1$
- $P$ of an infected group is determined by:
  1. whether and where the group is vaccinated
  2. age distribution of the group
- Specifically:
- $P_{A}^{UV}=\left( {Pasp}_{A}^{UV},{Pmm}_{A}^{UV},{Psc}_{A}^{UV},{Pdth}_{A}^{UV} \right)$ is the risk profile of the unvaccinated secondary cases in SG, derived from the age distribution of SG general population.
- $P_{A}^{V}=\left( {Pasp}_{A}^{V},{Pmm}_{A}^{V},{Psc}_{A}^{V},{Pdth}_{A}^{V} \right)$ is the risk profile of the vaccinated secondary cases in SG:

${Pasp}_{A}^{V}=1-\frac{1-\nu_{A}}{1-\mu_{A}}\times\left( 1-{Pasp}_{A}^{UV} \right)$

${Pmm}_{A}^{V}=\frac{1-\nu_{A}}{1-\mu_{A}}\times\left( 1-{Pasp}_{A}^{UV} \right)-\frac{1-\upsilon_{A}}{1-\mu_{A}}\times{(1-{Pasp}_{A}^{UV}-Pmm}_{A}^{UV})$

${Psc}_{A}^{V}=\frac{1-\upsilon_{A}}{1-\mu_{A}}\times{Psc}_{A}^{UV}$

${Pdth}_{A}^{V}=\frac{1-\upsilon_{A}}{1-\mu_{A}}\times{Pdth}_{A}^{UV}$

- $P_{AB}^{UV}=\left( {Pasp}_{AB}^{UV},{Pmm}_{AB}^{UV},{Psc}_{AB}^{UV},{Pdth}_{AB}^{UV} \right)$ is the risk profile of the unvaccinated returning Singaporean cases, whose age distribution is proxied by the age distribution of inbound travellers to SG.
- $P_{AB}^{V}=\left( {Pasp}_{AB}^{V},{Pmm}_{AB}^{V},{Psc}_{AB}^{V},{Pdth}_{AB}^{V} \right)$ is the risk profile of the vaccinated returning Singaporean:

${Pasp}_{AB}^{V}=1-\frac{1-\nu_{A}}{1-\mu_{A}}\times\left( 1-{Pasp}_{AB}^{UV} \right)$

${Pmm}_{AB}^{V}=\frac{1-\nu_{A}}{1-\mu_{A}}\times\left( 1-{Pasp}_{AB}^{UV} \right)-\frac{1-\upsilon_{A}}{1-\mu_{A}}\times{(1-{Pasp}_{AB}^{UV}-Pmm}_{AB}^{UV})$

${Psc}_{AB}^{V}=\frac{1-\upsilon_{A}}{1-\mu_{A}}\times{Psc}_{AB}^{UV}$

${Pdth}_{AB}^{V}=\frac{1-\upsilon_{A}}{1-\mu_{A}}\times{Pdth}_{AB}^{UV}$

- $P_{BA}^{UV}=\left( {Pasp}_{BA}^{UV},{Pmm}_{BA}^{UV},{Psc}_{BA}^{UV},{Pdth}_{BA}^{UV} \right)$ is the risk profile of the unvaccinated inbound travellers.
- $P_{BA}^{V}=\left( {Pasp}_{BA}^{V},{Pmm}_{BA}^{V},{Psc}_{BA}^{V},{Pdth}_{BA}^{V} \right)$ is the risk profile of the vaccinated inbound travellers:

${Pasp}_{BA}^{V}=1-\frac{1-\nu_{B}}{1-\mu_{B}}\times\left( 1-{Pasp}_{BA}^{UV} \right)$

${Pmm}_{BA}^{V}=\frac{1-\nu_{B}}{1-\mu_{B}}\times\left( 1-{Pasp}_{BA}^{UV} \right)-\frac{1-\upsilon_{B}}{1-\mu_{B}}\times{(1-{Pasp}_{BA}^{UV}-Pmm}_{BA}^{UV})$

${Psc}_{BA}^{V}=\frac{1-\upsilon_{B}}{1-\mu_{B}}\times{Psc}_{BA}^{UV}$

${Pdth}_{BA}^{V}=\frac{1-\upsilon_{B}}{1-\mu_{B}}\times{Pdth}_{BA}^{UV}$

- ${TC}_{A}^{UV}=({TCasp}_{A}^{UV},{TCmm}_{A}^{UV},{TCsc}_{A}^{UV})$ is the cost of managing an unvaccinated COVID patient at each severity level in SG, while ${TC}_{A}^{V}=({TCasp}_{A}^{V},{TCmm}_{A}^{V},{TCsc}_{A}^{V})$ is that for a vaccinated COVID patient
  - $T{Casp}_{A}$ is the cost of managing an asymptomatic case in SG
  - ${TCmm}_{A}$ is the cost of managing a case with mild/moderate symptoms in SG
  - ${TCsc}_{A}$ is the cost of managing a case with severe/critical symptoms in SG

1. (-) Cost of testing close contacts of secondary cases

$$-\left[ \begin{matrix} \left( {m1}_{BA}^{UV}+{m1}_{BA}^{V} \right) \\ +\left( {ud1s}_{BA}^{UV}+{ud1s}_{BA}^{V} \right) \end{matrix} \right]\times\eta_{A}$$

- $\eta_{A}$ is the testing cost on close contacts associated with one more case in SG
- ${ud1s}_{BA}^{UV}$ is number of unvaccinated cases travelling to Singapore, missed by country A fence but later develop symptoms
- ${ud1s}_{BA}^{V}$ is number of vaccinated cases travelling to Singapore, missed by country A fence but later develop symptoms

$${ud1s}_{BA}^{UV}={ud1}_{BA}^{UV}\times{Puds}_{BA}^{UV}$$

$${ud1s}_{BA}^{V}={ud1}_{BA}^{V}\times{Puds}_{BA}^{V}$$

- $\nu_{B}$ is the vaccine efficacy against symptomatic infection in rest of world. Assume $\nu_{B}=\nu_{A}$ in the base case analysis (This might be optimistic, as SG is using vaccines with best efficacy)

1. (-) Productivity loss due to COVID-19 morbidity and mortality in secondary cases

$$-\left[ {m1}_{BA}^{UV}+{m1}_{BA}^{V} \right]\times\omega_{AA}$$

- $\boldsymbol{\omega}\left( \boldsymbol{P,}\boldsymbol{L}_{\boldsymbol{K}}\boldsymbol{,}\boldsymbol{LD}_{\boldsymbol{J}} \right)$ is the expected productivity loss associated with one case from country J and treated at country K, whose probabilities of being asymptomatic/ mild/ moderate/ severe/ critical/ dead are $P$.

$\omega\left( P,L_{K},{LD}_{J} \right)$

$$=\varepsilon\left( \left( Pasp,Pmm,Psc,Pdth \right),\left( {Lasp}_{K},{Lmm}_{K},{Lsc}_{K} \right),{LD}_{J} \right)$$

$$=(Pasp\times{Lasp}_{K}+Pmm\times{Lmm}_{K}+Psc\times{Lsc}_{K}+Pdth\times{LD}_{J})\times{MInc}_{J}$$

- In this model, we only count Singaporean (returning Singaporean and local secondary cases), and we assume productivity loss is the same no matter where the Singaporean is treated. Therefore, we only have $L_{A}$, ${LD}_{A}$ and ${MInc}_{A}$.
- $L_{A}=\left( {Lasp}_{A},{Lmm}_{A},{Lsc}_{A} \right)$, varying between vaccinated and unvaccinated patients, in which
  - ${Lasp}_{A}$ is the productivity loss of an asymptomatic case being isolated in terms of patient-month
  - ${Lmm}_{A}$ is the productivity loss of a case with mild/ moderate symptoms being treated and isolated in terms of patient-month
  - ${Lsc}_{A}$ is the productivity loss of a case with severe/ critical symptoms being treated and isolated in terms of patient-month
- ${LD}_{A}$ is the productivity loss if a Singaporean case dies in terms of patient-month
- ${MInc}_{A}$ is monthly income of Singapore population
- $\omega_{AA}$ is the expected productivity loss of a secondary case treated at SG
  $\omega_{AA}=\frac{1-\chi_{A}}{1-\chi_{A}\times\mu_{A}}\omega\left( P_{A}^{UV},L_{A}^{UV},{LD}_{A} \right)+\frac{\chi_{A}-\chi_{A}\times\mu_{A}}{1-\chi_{A}\times\mu_{A}}\omega\left( P_{A}^{V},L_{A}^{V},{LD}_{A} \right)$
  - $\mu_{A}$ is the infection-blocking efficacy of vaccine used in SG, as defined in item 4.
  - $\chi_{A}$ is the vaccine coverage in SG, as defined in item 4.

1. (-) Health loss due to COVID-19 morbidity and mortality in secondary cases

$$-({m1}_{BA}^{UV}+{m1}_{BA}^{V})\times{Qaly}_{AA}\times{CET}_{A}$$

- $\boldsymbol{Qaly(P,Q)}$ is the expected QALY loss associated with one case, whose probabilities of being asymptomatic/ mild/ moderate/ severe/ critical/ dead are $\boldsymbol{P}$,and QALY loss associated with different states are $\boldsymbol{Q}$. $\boldsymbol{P}$ and $\boldsymbol{Q}$ differentiate between unvaccinated and vaccinated travellers.

$$Qaly\left( P,Q \right)$$

$$=Qaly\left( \left( Pasp,Pmm,Psc,Pdth \right), (Qasp,Qmm,Qsc,Qdth) \right)$$

$$=Pasp\times Qasp+Pmm\times Qmm+Psc\times Qsc+Pdth\times Qdth$$

- $Q=\left( Qasp,Qmm,Qsc, Qdth \right)$, varying between vaccinated and unvaccinated patients, in which
  - $Qasp$ is the QALY loss of an asymptomatic case
  - $Qmm$ is the QALY loss of a case with mild/ moderate symptoms
  - $Qsc$ is the QALY loss of a case with severe/ critical symptoms
  - $Qdth$ is the QALY loss of death
- ${Qaly}_{AA}$ is the expected QALY loss of a secondary case in SG

${Qaly}_{AA}=\frac{1-\chi_{A}}{1-\chi_{A}\times\mu_{A}}Qaly\left( P_{A}^{UV},Q^{UV} \right)+\frac{\chi_{A}-\chi_{A}\times\mu_{A}}{1-\chi_{A}\times\mu_{A}}Qaly\left( P_{A}^{V},Q^{V} \right)$

- ${CET}_{A}$ is the cost-effectiveness threshold of SG

## Net benefit: Returning travellers

1. (-) Cost of testing to enter Singapore

$$-\left[ \begin{matrix} E_{B}\times cART\times n0ART\left( t_{BA}^{UV} \right)+cART\times n1ART\left( t_{BA}^{UV} \right)+cART\times n2ART\left( t_{BA}^{UV} \right)+cART\times n3ART\left( t_{BA}^{UV} \right) \\ +E_{B}\times r0PCR\times n0PCR\left( t_{BA}^{UV} \right)+c1PCR\times n1PCR\left( t_{BA}^{UV} \right)+c2PCR\times n2PCR\left( t_{BA}^{UV} \right)+c3PCR\times n3PCR\left( t_{BA}^{UV} \right) \end{matrix} \right]\times N_{AB}^{UV}$$

$$-\left[ \begin{matrix} E_{B}\times c0ART\times n0ART\left( t_{BA}^{V} \right)+cART\times n1ART\left( t_{BA}^{V} \right)+cART\times n2ART\left( t_{BA}^{V} \right)+cART\times n3ART\left( t_{BA}^{V} \right) \\ +E_{B}\times r0PCR\times n0PCR\left( t_{BA}^{V} \right)+c1PCR\times n1PCR\left( t_{BA}^{V} \right)+c2PCR\times n2PCR\left( t_{BA}^{V} \right)+c3PCR\times n3PCR\left( t_{BA}^{V} \right) \end{matrix} \right]\times N_{AB}^{V}$$

- $E_{B}$ is the ratio of healthcare cost in the destination countries for Singaporean outbound travellers relative to that in Singapore
- $n0ART\left( t \right)$ is the number of ART pre-departure test per traveller, with value 0 or 1
- $n1ART\left( t \right)$ is the number of ART entry test per traveller, with value 0 or 1
- $n2ART\left( t \right)$ is the number of ART quarantine test per traveller
- $n3ART\left( t \right)$ is the number of ART exit test per traveller, with value 0 or 1
- $n0PCR\left( t \right)$ is the number of PCR pre-departure test per traveller, with value 0 or 1
- $n1PCR\left( t \right)$ is the number of PCR entry test per traveller, with value 0 or 1
- $n2PCR\left( t \right)$ is the number of PCR quarantine test per traveller
- $n3PCR\left( t \right)$ is the number of PCR exit test per traveller, with value 0 or 1
- $r0ART$ is the unit price of ART pre-departure test
- $r0PCR$ is the unit price of PCR pre-departure test
- $cART$ is the cost per ART test
- $c1PCR$ is the cost per PCR entry test
- $c2PCR$ is the cost per PCR quarantine test
- $c3PCR$ is the cost per PCR exit test

1. (-) Productivity loss due to quarantine

$$-N_{AB}^{UV}\times\rho_{A0}\times q_{A}^{UV}-N_{AB}^{V}\times\rho_{A0}\times q_{A}^{V}$$

- $\rho_{A0}$ is the daily productivity loss of quarantine a Singaporean

1. (-) Cost of managing returning travellers diagnosed by pre-departure test

$$-{d00}_{AB}^{UV}\times\varepsilon\left( P_{AB}^{UV},{TC}_{A}^{UV} \right){\times E}_{B}-{d00}_{AB}^{V}\times\varepsilon\left( P_{AB}^{V},{TC}_{A}^{V} \right){\times E}_{B}$$

2. (-) Cost of managing returning travellers diagnosed on arrival or during quarantine

$$-\left[ \begin{matrix} \left( {d2}_{AB}^{UV}+{ud2}_{AB}^{UV} \right)\times\varepsilon\left( P_{AB}^{UV},{TC}_{A}^{UV} \right) \\ -{ud2}_{AB}^{UV}\times\varepsilon\left( \tilde{P}_{AB}^{UV},{TC}_{A}^{UV} \right) \\ -{ud2}_{AB}^{UV}\times\varepsilon\left( \bar{P}_{AB}^{UV},{TC}_{A}^{UV} \right) \end{matrix} \right]-\left[ \begin{matrix} \left( {d2}_{AB}^{V}+{ud2}_{AB}^{V} \right)\times\varepsilon\left( P_{AB}^{V},{TC}_{A}^{V} \right) \\ -{ud2}_{AB}^{V}\times\varepsilon\left( \tilde{P}_{AB}^{V},{TC}_{A}^{V} \right) \\ -{ud2}_{AB}^{V}\times\varepsilon\left( \bar{P}_{AB}^{V},{TC}_{A}^{V} \right) \end{matrix} \right]$$

- $\tilde{P}$ is the risk profile with probability of being asymptomatic adjusted to be zero and probability of being symptomatic adjusted to be lower, i.e.

$$\tilde{P}=\left( \tilde{Pasp},\tilde{Pmm},\tilde{Psc},\tilde{Pdth} \right)$$

$$=\left( 0,Pmm,Psc,Pdth \right)\times\frac{Puds}{1-Pasp}$$

- $\bar{P}$ is the risk profile with probability of being symptomatic adjusted to be zero, i.e.

$$\bar{P}=\left( 1-Puds,0,0,0 \right)$$

1. (-) Cost of managing returning cases missed on arrival or during quarantine but develop symptoms afterwards

$$-{ud2}_{AB}^{UV}\times\varepsilon\left( \tilde{P}_{AB}^{UV},{TC}_{A}^{UV} \right)-{ud2}_{AB}^{V}\times\varepsilon\left( \tilde{P}_{AB}^{V},{TC}_{A}^{V} \right)$$

2. (-) Cost of managing secondary cases caused by infectious travellers who are not detected on arrival or during quarantine

$$-({m2}_{AB}^{UV}+{m2}_{AB}^{V})\times\varepsilon_{AA}$$

3. (-) Cost of testing close contacts of secondary cases and returning travellers missed on arrival or during quarantine but develop symptoms afterwards

$$-\left[ \begin{matrix} \left( {m2}_{AB}^{UV}+{m2}_{AB}^{V} \right) \\ +\left( {ud2s}_{AB}^{UV}+{ud2s}_{AB}^{V} \right) \end{matrix} \right]\times\eta_{A}$$

- $\eta_{A}$ is the testing cost associated with one more case in Singapore as introduced before
- ${ud2s}_{AB}^{UV}$ is number of unvaccinated cases returning to Singapore, missed by Singapore fence but later develop symptoms
- ${ud2s}_{AB}^{V}$ is number of vaccinated cases returning to Singapore, missed by Singapore fence but later develop symptoms

$${ud2s}_{AB}^{UV}={ud2}_{AB}^{UV}\times(1-{Pasp}_{AB}^{UV})$$

$${ud2s}_{AB}^{V}={ud2}_{AB}^{V}\times\left( 1-{Pasp}_{AB}^{V} \right)$$

1. (-) Productivity loss due to COVID-19 morbidity and mortality

$$-\left[ {m2}_{AB}^{UV}+{m2}_{AB}^{V} \right]\times\omega_{AA}$$

$$-\left( {d00}_{AB}^{UV}+{d2}_{AB}^{UV}+{ud2}_{AB}^{UV} \right)\times\omega\left( P_{AB}^{UV},L_{AB}^{UV},{LD}_{A} \right)$$

$$-\left( {d00}_{AB}^{V}+{d2}_{AB}^{V}+{ud2}_{AB}^{V} \right)\times\omega\left( P_{AB}^{V},L_{AB}^{V},{LD}_{A} \right)$$

$$+{ud2}_{AB}^{UV}\times\omega\left( \bar{P}_{AB}^{UV},L_{AB}^{UV},{LD}_{A} \right)$$

$$+{ud2}_{AB}^{V}\times\omega\left( \bar{P}_{AB}^{V},L_{AB}^{V},{LD}_{A} \right)$$

2. (-) Health loss due to COVID-19 morbidity and mortality

$$-\left[ \begin{aligned} \begin{matrix} \left( {m2}_{AB}^{UV}+{m2}_{AB}^{V} \right)\times{Qaly}_{AA} \\ +\left( {{d00}_{AB}^{UV}+d2}_{AB}^{UV}+{ud2}_{AB}^{UV} \right)\times Qaly\left( P_{AB}^{UV},Q^{UV} \right) \\ -{ud2}_{AB}^{UV}\times Qaly\left( \bar{P}_{AB}^{UV},Q^{UV} \right) \end{matrix} \\ +\left( {{d00}_{AB}^{V}+d2}_{AB}^{V}+{ud2}_{AB}^{V} \right)\times Qaly\left( P_{AB}^{V},Q^{V} \right) \\ -{ud2}_{AB}^{V}\times Qaly\left( \bar{P}_{AB}^{V},Q^{V} \right) \end{aligned} \right]\times{CET}_{A}$$

## Other outcomes

1. Number of imported cases: Inbound travellers

$${d1}_{BA}^{UV}+{ud1}_{BA}^{UV}\times{Puds}_{BA}^{UV}$$

$$+{d1}_{BA}^{V}+{ud1}_{BA}^{V}\times{Puds}_{BA}^{V}$$

2. Number of imported cases: Returning travellers

$${d2}_{AB}^{UV}+{ud2}_{AB}^{UV}\times{Puds}_{AB}^{UV}$$

$$+{d2}_{AB}^{V}+{ud2}_{AB}^{V}\times{Puds}_{AB}^{V}$$

3. Number of secondary cases: Inbound travellers

$${m1}_{BA}^{UV}+{m1}_{BA}^{V}$$

4. Number of secondary cases: Returning travellers

$${m2}_{AB}^{UV}+{m2}_{AB}^{V}$$

5. Number of deaths in inbound travellers who finally come to Singapore

$$\left( N_{BA}^{UV}\times{Prev}_{BA}^{UV}-{d0}_{BA}^{UV} \right)\times{Pdth}_{BA}^{UV}$$

$$+\left( N_{BA}^{V}\times{Prev}_{BA}^{V}-{d0}_{BA}^{V} \right)\times{Pdth}_{BA}^{V}$$

6. Number of deaths in secondary cases caused by inbound travellers

$$({m1}_{BA}^{UV}+{m1}_{BA}^{V})\times{Pdth}_{AA}$$

in which

$${Pdth}_{AA}=\frac{1-\chi_{A}}{1-\chi_{A}\times\mu_{A}}{Pdth}_{A}^{UV}+\frac{\chi_{A}-\chi_{A}\times\mu_{A}}{1-\chi_{A}\times\mu_{A}}{Pdth}_{A}^{V}$$

1. Number of deaths in returning travellers

$$\left( N_{AB}^{UV}\times{Prev}_{AB}^{UV} \right)\times{Pdth}_{AB}^{UV}$$

$$+\left( N_{AB}^{V}\times{Prev}_{AB}^{V} \right)\times{Pdth}_{AB}^{V}$$

2. Number of deaths in secondary cases caused by returning travellers

$$({m1}_{AB}^{UV}+{m1}_{AB}^{V})\times{Pdth}_{AA}$$

3. Number of critical cases in inbound travellers

$$(N_{BA}^{UV}\times{Prev}_{BA}^{UV}-{d0}_{BA}^{UV})\times{Pc}_{BA}^{UV}$$

$$+(N_{BA}^{V}\times{Prev}_{BA}^{V}-{d0}_{BA}^{V})\times{Pc}_{BA}^{V}$$

4. Number of critical cases in secondary cases caused by inbound travellers

$$({m1}_{BA}^{UV}+{m1}_{BA}^{V})\times{Pc}_{AA}$$

in which

$${Pc}_{AA}=\frac{1-\chi_{A}}{1-\chi_{A}\times\mu_{A}}{Pc}_{A}^{UV}+\frac{\chi_{A}-\chi_{A}\times\mu_{A}}{1-\chi_{A}\times\mu_{A}}{Pc}_{A}^{V}$$

1. Number of critical cases in returning travellers

$$\left( N_{AB}^{UV}\times{Prev}_{AB}^{UV}-{d00}_{AB}^{UV} \right)\times{Pc}_{AB}^{UV}$$

$$+\left( N_{AB}^{V}\times{Prev}_{AB}^{V}-{d00}_{AB}^{V} \right)\times{Pc}_{AB}^{V}$$

2. Number of critical cases in secondary cases caused by returning travellers

$$({m1}_{AB}^{UV}+{m1}_{AB}^{V})\times{Pc}_{AA}$$
